# Prevalence of Anxiety and Depression Among the General Population in Africa During the COVID-19 Pandemic: A Systematic Review and Meta-Analysis

**DOI:** 10.3389/fpubh.2022.814981

**Published:** 2022-05-17

**Authors:** Umar Muhammad Bello, Priya Kannan, Muhammad Chutiyami, Dauda Salihu, Allen M. Y. Cheong, Tiev Miller, Joe Wing Pun, Abdullahi Salisu Muhammad, Fatima Ado Mahmud, Hussaina Abubakar Jalo, Mohammed Usman Ali, Mustapha Adam Kolo, Surajo Kamilu Sulaiman, Aliyu Lawan, Isma'il Muhammad Bello, Amina Abdullahi Gambo, Stanley John Winser

**Affiliations:** ^1^Centre for Eye and Vision Research Limited, Hong Kong, Hong Kong SAR, China; ^2^Department of Physiotherapy, Yobe State University Teaching Hospital, Damaturu, Nigeria; ^3^Department of Rehabilitation Sciences, The Hong Kong Polytechnic University, Hong Kong, Hong Kong SAR, China; ^4^School of Nursing, Institute of Health and Management, Sydney, NSW, Australia; ^5^School of Nursing, The Hong Kong Polytechnic University, Hong Kong, Hong Kong SAR, China; ^6^School of Optometry, The Hong Kong Polytechnic University, Hong Kong, Hong Kong SAR, China; ^7^Physiotherapy Department, Yobe State Specialist Hospital, Damaturu, Nigeria; ^8^Department of Paediatrics, Yobe State Specialist Hospital, Damaturu, Nigeria; ^9^Department of Rehabilitation Sciences, University of Maiduguri, Maiduguri, Nigeria; ^10^Department of Geography, University of Maiduguri, Maiduguri, Nigeria; ^11^Department of Physiotherapy, Bayero University Kano, Kano, Nigeria; ^12^Department of Rehabilitation Science, Western University, London, ON, Canada; ^13^Department of Medicine and Surgery, Ahmadu Bello University, Zaria, Nigeria; ^14^Department of Paediatrics, Barau Dikko Teaching Hospital, Kaduna, Nigeria

**Keywords:** Africa, COVID-19, pandemics, anxiety, depression

## Abstract

**Background:**

Medical and socio-economic uncertainties surrounding the COVID-19 pandemic have had a substantial impact on mental health. This study aimed to systematically review the existing literature reporting the prevalence of anxiety and depression among the general populace in Africa during the COVID-19 pandemic and examine associated risk factors.

**Methods:**

A systematic search of the following databases African Journal Online, CINAHL, PubMed, Scopus, and Web of Science was conducted from database inception until 30th September 2021. Studies reporting the prevalence of anxiety and/or depression among the general populace in African settings were considered for inclusion. The methodological quality of included studies was assessed using the Agency for Healthcare Research and Quality (AHRQ). Meta-analyses on prevalence rates were conducted using Comprehensive Meta-analysis software.

**Results:**

Seventy-eight primary studies (62,380 participants) were identified from 2,325 studies via electronic and manual searches. Pooled prevalence rates for anxiety (47%, 95% CI: 40–54%, *I*^2^ = 99.19%) and depression (48%, 95% CI: 39–57%, *I*^2^ = 99.45%) were reported across Africa during the COVID-19 pandemic. Sex (female) and history of existing medical/chronic conditions were identified as major risk factors for anxiety and depression.

**Conclusions:**

The evidence put forth in this synthesis demonstrates the substantial impact of the pandemic on the pervasiveness of these psychological symptoms among the general population. Governments and stakeholders across continental Africa should therefore prioritize the allocation of available resources to institute educational programs and other intervention strategies for preventing and ameliorating universal distress and promoting psychological wellbeing.

**Systematic Review Registration:**

https://www.crd.york.ac.uk/prospero/display_record.php?ID=CRD42021228023, PROSPERO CRD42021228023.

## Introduction

Coronavirus disease-2019 (COVID-19), a highly transmissible ailment caused by SARS-CoV-2 virus, has generated unparalleled distress on a global scale since it was first diagnosed in Wuhan City in mainland China, with the earliest symptoms reported on the 1st of December 2019 (1, 2). COVID-19 was declared a pandemic by the World Health Organization (WHO) on the 11th of March 2020 (1, 3), thus joining in a series of historic global outbreaks/pandemics [including Athenian plague of 430 B.C., Antonine plague of 165–180 A.D., Justinian plague, Black death plague, Human Immunodeficiency Virus (HIV) pandemic, Smallpox outbreak of 1972, Severe Acute Respiratory Syndrome (SARS), Swine flu pandemic of 2009, Ebola outbreak of 2014–2016, and Zika outbreak of 2015–2016] that resulted in millions of human deaths (1, 4). Over 200 million cases and about 4.5 million COVID-19-related mortalities have been reported across the globe (3). Despite the concerted efforts of Governments and stakeholders to ameliorate the threat of the disease, incidence and mortality rates continue to increase across different countries and territories. Other factors such as the rapid mutation of the virus exacerbate the level of distress experienced on a global scale (3, 5). Uniquely, the COVID-19 pandemic not only affects global health systems but also severely affects the global economy and financial markets (6). A palpable decline in income, greater unemployment, and on-going disruptions in the transportation, service, and manufacturing industries are among the many consequences of the current pandemic (6).

The African continent is comprised of 54 countries situated across five distinct regions (Eastern, Middle, Northern, Southern and Western) and accounts for ~17.51% of all global inhabitants (i.e., 1.2 billion) as of 2021 (7, 8). Many countries across the continent are faced with healthcare problems due to weak healthcare infrastructures and socio-economic challenges (9). Broadly, these challenges arise from inadequate human resources, poor leadership and management, and inadequate budgetary allocation to cover essential healthcare expenditures (9). Other indirect factors limiting the provision of and access to quality healthcare in most African countries include poverty, conflict, unemployment, food insecurity, inequality, climate change, and rapid industrialization (10). Given the peculiar nature of the disease and the subsequent initiatives to contain its spread, the current pandemic has had a direct impact on the overall wellbeing of the global populace (11). Factors such as high transmissibility, a remarkable number of hospital admissions, the need for isolation during treatment, respiratory problems, and increased morbidity and mortality rates, have led to a general decline in overall health and wellbeing (3). Compared to countries in other parts of the world with robust and sophisticated healthcare systems, many African countries are faced with compounded challenges due to existing deficiencies in healthcare service delivery (12). Other indirect factors associated with the pandemic such as travel restrictions, lockdowns, job loss and economic decline, although affecting the entire global population, may have a relatively greater impact on Africans (12). These multifactorial and often indirect consequences of the pandemic not only reduce quality of life, and general wellbeing, but also affect mental health status at the societal level, leading to the pervasiveness of symptoms such as anxiety and depression (13). Therefore, a summative examination of the mental health status in the African population during the pandemic is of paramount importance.

A growing number of primary studies have been conducted to ascertain data relating to the effect of the COVID-19 pandemic on various mental health domains across the globe (14, 15). Several systematic reviews and meta-analyses have also been conducted to summarize rising mental health concerns on both a regional and global scale (13, 16–18). Other systematic reviews have examined the effect of the COVID-19 pandemic on the mental health of children and adolescents (19), and the prevalence of antenatal and postnatal anxiety and depressive symptoms among pregnant women (20). The prevalence of anxiety, depression, and other psychological variables in the general population during the pandemic have also been examined (21). However, an assessment of the prevalence of these symptoms and associated risk factors among the general populace in Africa is currently lacking from the literature. Therefore, the aim of this systematic review and meta-analysis was to address this knowledge gap by examining the prevalence of anxiety and depression among the general populace across the African continent during the COVID-19 pandemic, and to assess the associated risk factors as a secondary aim of the study.

## Methods

This systematic review was conducted in accordance with the Preferred Reporting Items for Systematic Reviews and Meta-Analyses (PRISMA) guidelines (22). A protocol of the study was first registered with the International Prospective Register of Systematic Reviews (PROSPERO; Ref. No: CRD42021228023) in January of 2021 prior to commencement. Authors (SJW, UMB, MC, DS, PK, and AMYC) conceptualized the study, and developed the protocol.

### Search Strategy

A systematic search of the following databases African Journal Online, CINAHL, PubMed, Scopus, and Web of Science from database inception until 30th September 2021 was performed. The search terms were grouped under three themes, namely: “COVID-19,” “anxiety & depression,” and “Africa”. The search theme “Africa” included all the 54 African countries to ensure wider coverage of the studies conducted across the continent. The electronic search involved combining terms under each theme using the Boolean operator “OR”. The search themes were then combined using the Boolean “AND” (Appendix 1 presents the details of the search strategy adopted in the study). Citation management software (EndNote X9, Clarivate Analytics, Philadelphia, Pennsylvania, USA) was used to archive and organize search results and remove duplicates. Two of the authors (UMB, JWP) independently conducted the electronic search. Any discrepancies were resolved by consulting a third author (SJW). The reference lists of included studies were manually searched by four review authors (DS, HAJ, AAG, and IMB).

### Study Eligibility Criteria

Studies that adopted a survey method of data collection were included if they (1) assessed anxiety and/or depression among the general populace in African settings, during the COVID-19 pandemic and (2) were available in full text. Gray literature (unpublished studies) were included to minimize publication bias as recommended by Paez (23). Excluded studies were (1) systematic reviews; (2) review protocols; (3) case reports; (4) case series (5) qualitative papers; (6) editorials or (7) conference abstracts.

### Article Screening

Studies identified through the electronic search underwent a three-stage title, abstract and full text screening. Two of the authors (SJW, UMB) independently selected eligible studies that adopted a survey methodology to examine the prevalence of anxiety and/or depression among the general populace in Africa during the COVID-19 pandemic. Any discrepancies were resolved by consulting a third author (PK).

### Data Extraction

The primary data extracted from included studies were the prevalence rates (summary data) of anxiety and/or depression that were assessed using questionnaires in the included studies. Other data extracted included associated risk factors, author details, aims and objectives, sample size, participant sex and other demographic characteristics, survey instrument details, other psychological variable(s) assessed, country of origin and overall conclusions. Data extraction was conducted independently by two authors (ASM, FAM) using a standardized tool designed in Excel. Disagreements between authors during the data extraction process were resolved by further discussions with a third author (UMB).

### Quality Appraisal of the Included Studies

The methodological quality of the included studies was assessed using the Agency for Healthcare Research and Quality (AHRQ) checklist for observational studies (24). Two authors (TM, MUA) independently conducted the methodological quality assessment. The assessment criteria were adapted from a previous study (24). Scores ranged from 0 to 10, with higher scores indicating higher quality. An overall score ≥ 7 was indicative of good methodological quality across studies, while scores of 1–3 and 4–6 indicated poor and moderate quality, respectively.

### Data Synthesis and Statistical Analysis

The extracted data were first narratively synthesized (CM, DS, and MAK) due to considerable heterogeneity in reported outcomes across included studies. Risk factors for anxiety and depression reported in the included studies were also synthesized. The narrative synthesis of quantitative findings [i.e., expressed as percentages, correlations (*r*), between-group differences (χ^2^), odds ratios (OR) or adjusted odds ratios (AOR) with 95% confidence intervals (CI)] was conducted in accordance with recommendations provided by the Centre for Reviews and Dissemination (25). Using a random-effects model, reported prevalence estimates (i.e., percentages) for anxiety and/or depression were aggregated in several meta-analyses to generate pooled prevalence rates. The percentage prevalence score from each study was then converted to a raw prevalence score using the following equation: (overall population × reported percentage)/100. Meta-analyses were conducted by pooling the “raw prevalence score” and the “overall population score” from each study (https://www.meta-analysis.com/pages/tutorials.php). Two separate meta-analyses were conducted to estimate the overall effect of the COVID-19 pandemic on the prevalence of anxiety and depression in the African continent. Subsequent subgroup analyses were conducted by aggregating studies according to region (i.e., Eastern, Middle, Northern, Southern, and Western Africa), country (for subgroups of ≥ 2 studies with data originating from the same country), outcome measures, and study period. A random-effects model was choosen based on the high clinical (for example study population, study period, and gender distribution) and methodological (for example study designs and mode of data collection) heterogeniety among the studies included in the meta-analyses (26). All meta-analyses were conducted using Comprehensive Meta-analysis software (CMA version 3.0, Biostat Inc., Englewood, New Jersey, USA) (UMB, CM, and DS).

## Results

**Figure 1:**
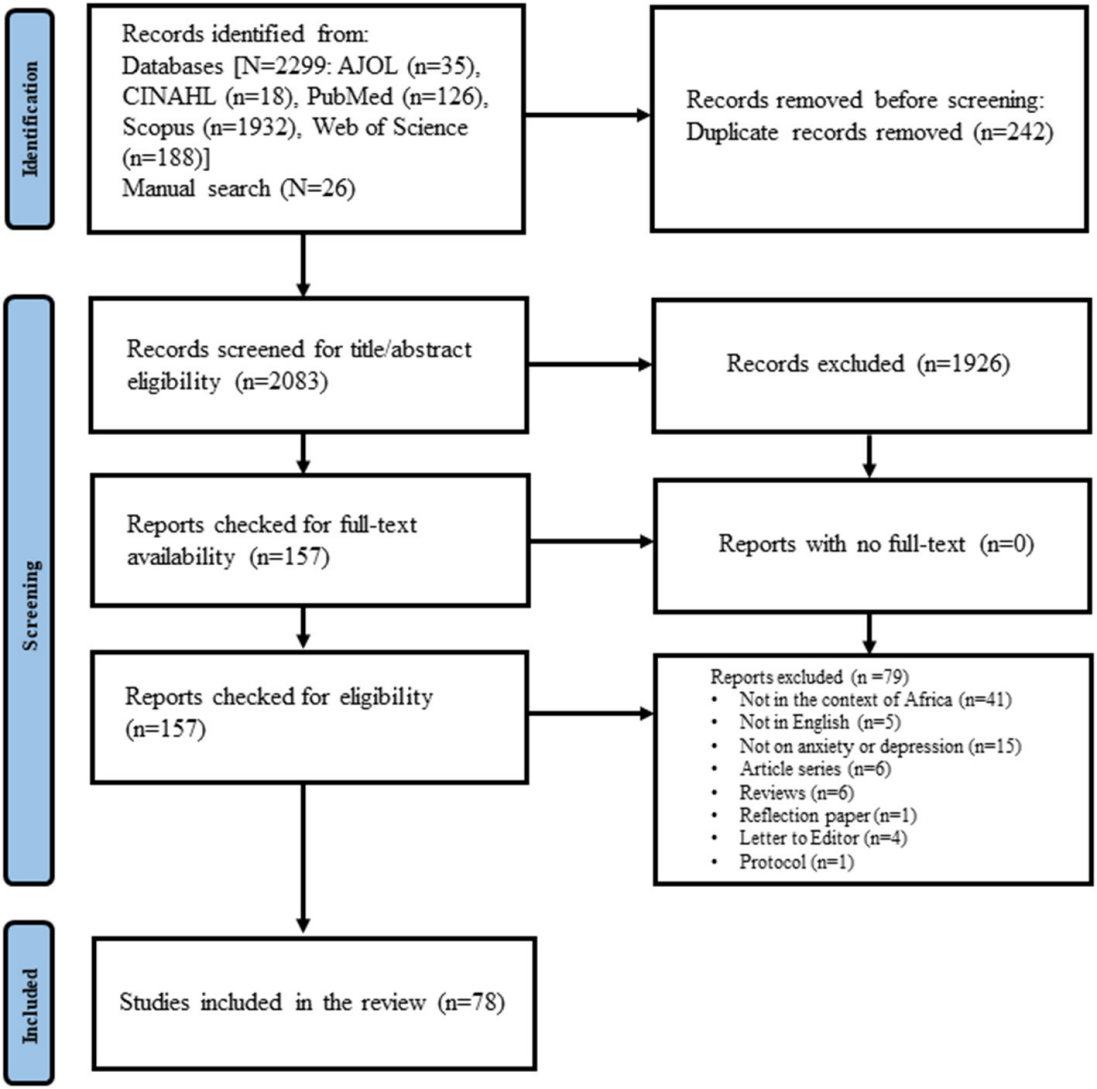
Study flowchart.

A total of 78 studies (14, 15, 26–−101) conducted between January 2020 and February 2021 were included in the final review (Figure 1). Five of these inclusions were categorized as gray literature (unpublished studies) (27–32).

### Characteristics of Included Studies

A total of 62,380 participants were recruited in the included studies, with the largest proportion of participants being male (49.8%, *n* = 31,074). Females constituted 47.3% of all participants (*n* = 29,479). Eight studies (2.9%, *n* = 1,854) did not report participant sex. More than half of participants were from the general population (69.9%, *n* = 43,646), Health workers constituted the second largest proportion (16%, *n* = 10,367). Others were students (11.6%, *n* = 7,249), and people with medical conditions (2.0%, *n* = 1,300). Except for Dyer et al. (33), who recruited participant samples aged 10–24 years, overall age ranged from 18 to 84 years. Only ten studies (12.8%) collected data using researcher-developed questionnaires. The majority (64.1%, *n* = 50) utilized instruments which were validated and reliable. However, only 24.4% (*n* = 19) of the studies used translated and cross-culturally validated assessments during data collection.

Many of the included studies were from Ethiopia (24.4%, *n* = 19), Nigeria (20.5%, *n* = 16), and Egypt (14.1%, *n* = 11). Others were from Libya (5.1%, *n* = 4), South Africa (3.8%, *n* = 3), Ghana (3.8%, *n* = 3), Uganda (2.6%, *n* = 2), Morocco (2.6%, *n* = 2), Kenya (2.6%, *n* = 2), Tunisia (2.6%, *n* = 2), Libya (2.6%, *n* = 2), Cameroon (2.6%, *n* = 2), Zambia (1.3%, *n* = 1), Algeria (1.3%, *n* = 1), Togo (1.3%, *n* = 1), Sudan (1.3%, *n* = 1), and Mali (1.3%, *n* = 1). Four studies (5.1%) covered more than one African country (15, 34–36), with two others (2.6%) covering sub-Saharan Africa (37, 38) and one other (1.3%) west African country (39).

The most commonly used tools for assessing anxiety and depression among the included studies were the Generalised Anxiety Disorder Assessment (GAD-7) (29.5%, *n* = 23), and Patient Health Questionnaire-9 (PHQ-9) (23.1%, *n* = 18), respectively. These were followed by the Depression, Anxiety and Stress Scale (DASS-21) (23.1%, *n* = 18), and the Hospital Anxiety and Depression Scale (HADS) (8.9%, *n* = 7). A summary of characteristics for included studies is provided in Supplementary Table 1.

### Methodological Quality

Most included studies [78% (*n* = 61)] were of good methodological quality (ARHQ ≥ 7). The median (range) score was 8 (5–10) points. The quality appraisal indicated that all included studies provided sources of information regarding the assessment of depression and anxiety, and participant recruitment settings (Appendix 3).

### Quantitative Synthesis on COVID-19 Related Anxiety in Africa

#### Overall Prevalence of Anxiety in Africa During the COVID-19 Pandemic

**Figure 2:**
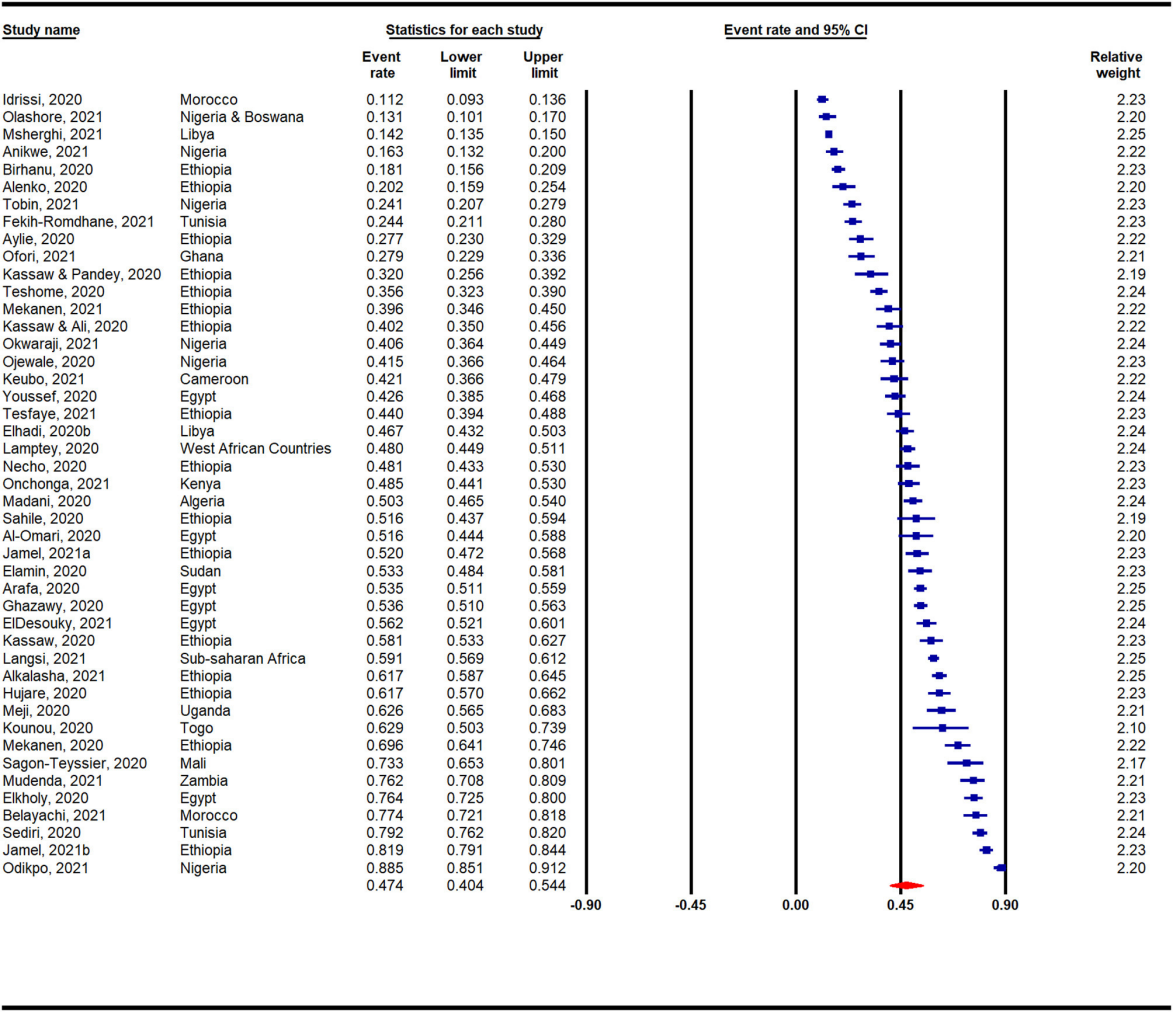
Overall prevalence of anxiety in Africa during the COVID-19 pandemic.

**Figure 3:**
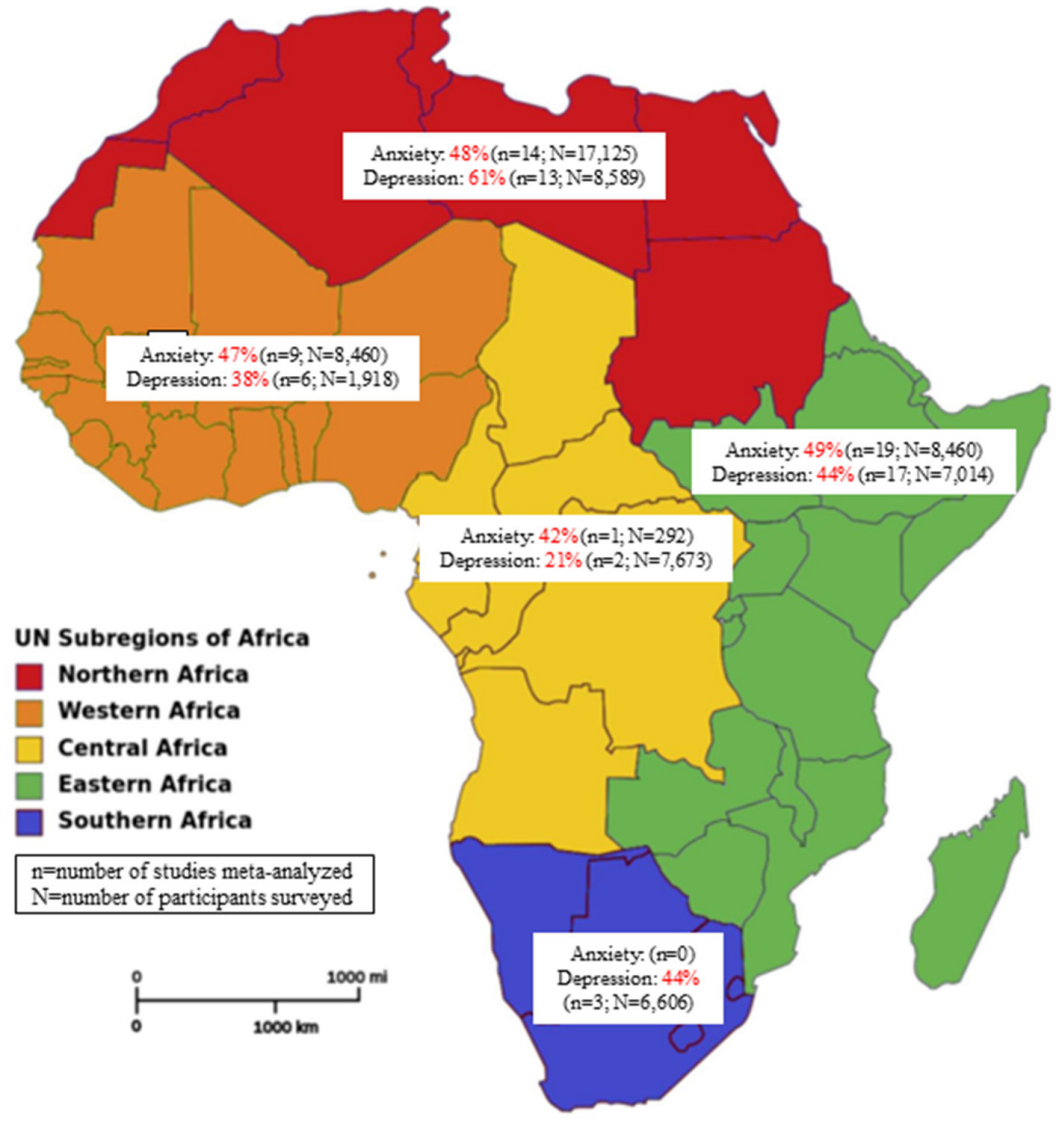
Prevalence of anxiety and depression in Africa by regions during the COVID-19 pandemic (source of image: File:Africa map regions 2.png-Wikipedia).

Figure 2 presents a meta-analysis on the prevalence of anxiety across Africa during the COVID-19 pandemic. A pooled prevalence rate of 47% (95% CI: 40–54%) was reported among 45 individual studies that surveyed 31,300 participants. Heterogeneity among the pooled studies was high (*I*^2^ = 99.19%, *p* < 0.001). The prevalence of anxiety in Africa during the COVID-19 pandemic according to region is presented in Appendix 3 and Figure 3. A forest plot illustrating further sub-analysis based on the outcome measures utilized to assess anxiety in the included studies is presented as Appendix 4.

#### Prevalence of Anxiety in Africa by Countries During the COVID-19 Pandemic

**Figure 4:**
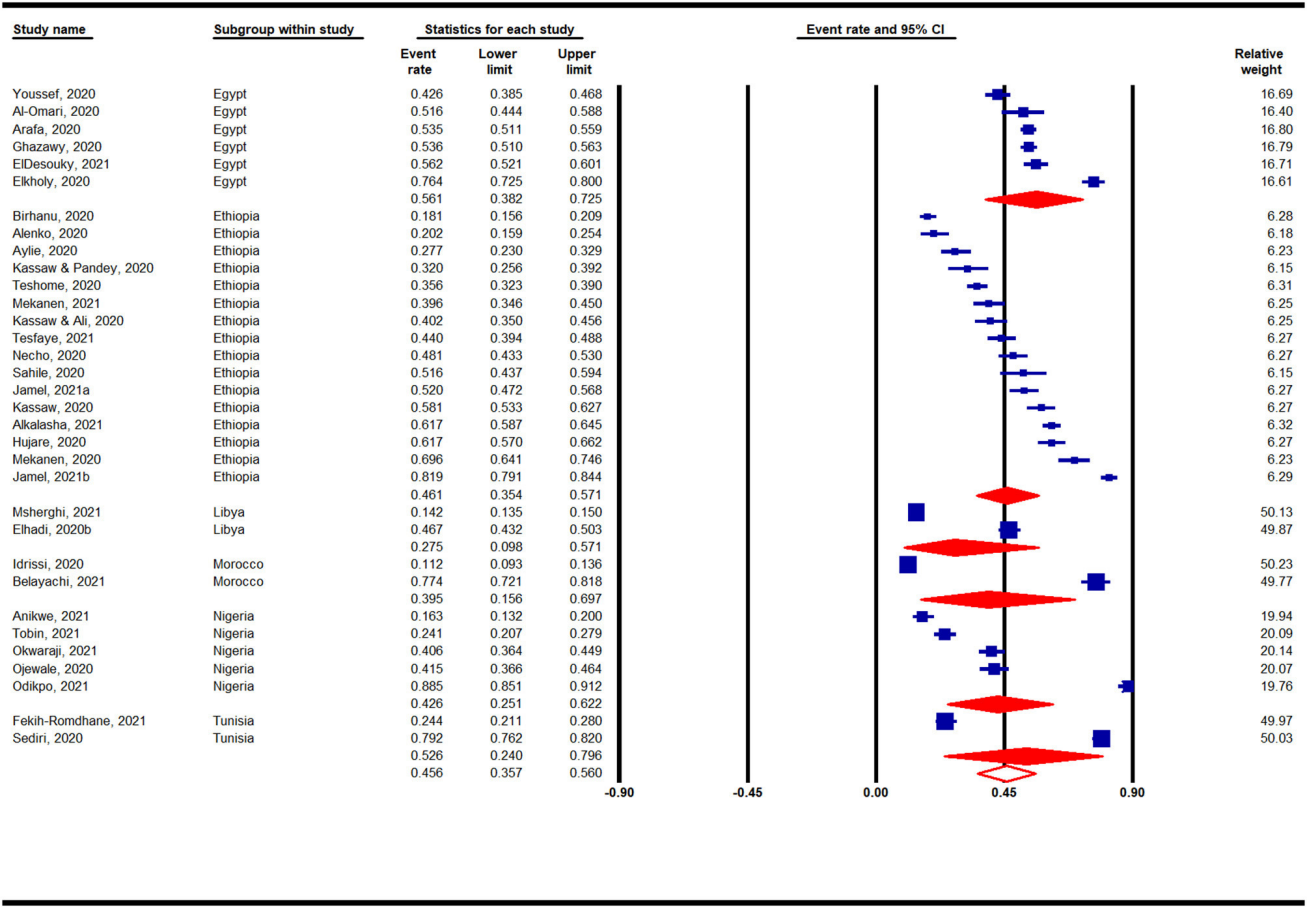
Prevalence of anxiety in Africa by countries during the COVID-19 pandemic.

Country-based subgroup analyses on the prevalence of anxiety in Africa during the COVID-19 pandemic indicated that among the 6 countries analyzed (with ≥ 2 studies) (Figure 4), the highest prevalence was reported for studies conducted in Egypt (56%, 95% CI: 38–73%, *n* = 6), whereas the lowest prevalence was reported for studies in Libya (28%, 95% CI: 10–57%, *n* = 2).

#### Prevalence of Anxiety in Africa by the Period of the COVID-19 Pandemic

Sub-analysis based on the period of the COVID-19 pandemic is presented as Appendix 5. The periods are categorized into Early 2020 (1st January−30th June), Late 2020 (1st July−31st Dec), and Early 2021 (1st January−30th June). Higher prevalence was reported for studies conducted in the Late 2020 (53%, 95% CI: 39–66%, *n* = 6) in comparison with Early 2020 (47%, 95% CI: 39–56%, *n* = 30).

### Narrative Synthesis on COVID-19 Related Anxiety in Africa

Overall, sixty-five studies (*n* = 65) assessed anxiety and its related symptoms in Africa (Table 1). Anxiety prevalence ranged from 11.3% (*n* = 827) among the general population in Morocco (81) to 88.5% (*n* = 418) among frontline nurses in Nigeria (87) (Table 1). DASS-21 (*n* = 16) and GAD-7 (*n* = 21) were the most used measures of anxiety, with reported proportions ranging from 18.1 to 81.9% and 24.1 to 76.4%, respectively. Anxiety prevalence was significantly associated with depression or depressive symptoms (35, 40, 50). Other anxiety-related symptoms reported were insomnia (14, 27, 40, 54, 60, 65, 67, 70, 72, 77, 81), fear of covid (29, 53, 92), psychological distress (30), worry (38), and fear of getting sick (93) (Table 1).

**Table 1:** Anxiety-related COVID-19 effect in Africa.

**Outcome**	**Measure**	**References**	**Effect of COVID-19 on the outcome**		**Effect size/comment**
			**Overall reported**	**No overall reported**	
Overall anxiety	ARS	(36)	√		13.1%, *n* = 373
prevalence	CAS	(40)	√		Mean (SD) = 56 (20.2), 95% CI: 15.9–25.3, *n* = 277
	CAS	(41)	√		16.3%, *n* = 460
	CIAS	(42)	√		18.1%, *n* = 801
	DASS-21	(43)	√		51.6%, *n* = 182
	DASS-21	(27)	√		53.5%, *n* = 1,629
	DASS-21	(44)	√		27.7%, *n* = 314
	DASS-21	(35)	√		Mean (SD) = 8.20 (9.00), *n* = 264 Algeria; 7.45 (8.13), *n* = 293 Egypt; 5.00 (7.24), *n* = 435 Nigeria.
	DASS-21	(45)	√		24.4%, *n* = 603
	DASS-21	(46)	√		53.6%, *n* = 1,335
	DASS-21	(47)	√		81.9%, *n* = 816
	DASS-21	(48)	√		44.4%, *n* = 420. The outcome was assessed as general mental health crises, comprising depression, anxiety, and stress
	DASS-21	(49)	√		58%, *n* = 420
	DASS-21	(50)	√		Mean (SD) = 14.44 (7.37)
	DASS-21	(51)	√		69.6%, *n* = 293
	DASS-21	(52)	√		39.6%, *n* = 338
	DASS-21	(53)	√		27.8%, *n* = 272
	DASS-21	(54)	√		42.6%, *n* = 540
	DASS-21	(55)	√		51.6%, *n* = 153
	DASS-21	(56)	√		79.2%, *n* = 751
	GAD-7	(57)		√	χ^2^ = 06.71; df = 1. Significantly higher among health professionals compared to the general population.
	GAD-7	(58)	√		53.3%, *n* = 396
	GAD-7	(37)	√		Mean (SE) = 7.23 (0.56), *n* = 83. Assessed for three African countries (South Africa, Egypt, Uganda)
	GAD-7	(59)	√		56.2%, *n* = 584
	GAD-7	(60)	√		52%, *n* = 417
	GAD-7	(61)	√		40.2%, *n* = 326
	GAD-7	(62)	√		32.2%, *n* = 178. Among women at perinatal service
	GAD-7	(63)	√		62.9%, *n* = 62
	GAD-7	(39)	√		48%, *n* = 1,000
	GAD-7	(64)	√		40.6%, *n* = 520
	GAD-7	(65)		√	χ^2^ = 0.08, df=1, *n* = 502. Assessed difference among gender.
	GAD-7	(66)	√		76.2%, *n* = 273
	GAD-7	(67)	√		48.1%, *n* = 403. Among people with disability
	GAD-7	(68)	√		48.5%, *n* = 476
	GAD-7	(69)	√		61.7%, *n* = 1,080
	GAD-7	(70)	√		73.3%, *n* = 135
	GAD-7	(71)	√		Mean (SD) = 7.2 (5.1), *n* = 2,430
	GAD-7	(72)	√		76.4%, *n* = 484
	GAD-7	(73)	√		14.2%, *n* = 8,084
	GAD-7	(74)	√		35.6%, *n* = 798
	GAD-7	(75)	√		24.1%, *n* = 543
	GHQ-12	(14)	√		Mean (SD) = 15.16 (4.97), *n* = 182
	HADS	(76)	√		42.2%, *n* = 292
	HADS	(28)	√		77.4%, *n* = 287
	HADS	(77)	√		Mean (SD) = 8.0 (4.5), *n* = 320
	HADS	(78)	√		Mean (SD) = 11.91 (3.81), *n* = 154
	HADS	(79)	√		61.8%, *n* = 423. Among chronic medical patients
	HADS	(32)	√		41.5%, *n* = 386
	HADS	(80)	√		46.7%, *n* = 745
	HARS	(81)	√		11.3%, *n* = 827
	HSCL	(15)	√		Mean (SD) = 0.82 (1.08), *n* = 626 Congo; 1.25 (1.25), *n* = 174 Rwanda and 0.63 (0.62), *n* = 242 Togo.
	ICC	(82)	√		50.3%, *n* = 678
	IES	(83)	√		Mean (SD) = 34.25 (15.0), *n* = 502. Assessed as total psychological impact (referenced to anxiety and stress)
	MINI	(84)	√		44%, *n* = 420
	Self-Questionnaire	(38)	√		59.1%, *n* = 2,005. Various African countries (not specified)
	Self-Questionnaire	(85)		√	OR = 1.26, 95% CI (0.429–3.72), *n* = 346. Anxiety assessed for risk of COVID-19 between male and female health workers. No overall anxiety reported
	Self-Questionnaire	(86)	√		62.7%, *n* = 254
	Self-Questionnaire	(87)	√		88.5%, *n* = 418
	Self-Questionnaire	(88)		√	Mean (SD) for males 8.203 (5.93) and females 10.14 (5.55), *n* = 183.
	Self-Questionnaire	(89)		√	Mean (SD) for males 3.653 (3.821) and females 6.6 (4.871), *n* = 69.
	Self-Questionnaire	(90)		√	Mean (SD) for males 7.568 (4.336) and females 10.59 (4.402), *n* = 287.
Anxiety-related	AIS	(81)		√	19.3%, *n* = 827
insomnia	DASS-21	(27)	√		23.1% prevalence of inadequate sleeping (<6 h/day), *n* = 1,629
	GHQ-12	(14)	√		Mean (SD) = 13.95(4.82), *n* = 182
	ISI	(72)	√		67.7%, *n* = 473
	ISI	(60)	√		28.3%, *n* = 417
	ISI	(65)		√	χ^2^ = 4.13, df = 3, *P* > 0.05. The assessed difference among gender. No overall estimate was reported.
	ISI	(70)	√		77%, *n* = 135
	ISS	(57)		√	χ^2^ = 40.21; df = 3. Significantly higher among health professionals compared to the general population.
	ISS	(67)	√		71%, *n* = 403
	ISS	(54)	√		51.9%, 540
Anxiety-related	DASS-21	(27)	√		48.8%, *n* = 1,629
stress	DASS	(44)	√		32.5%, *n* = 314
	DASS-21	(35)	√		Mean (SD) = 11.99 (10.63) Algeria; 12.31 (10.03) Egypt; 6.57 (8.04) Nigeria; *n* = 992.
	DASS-21	(45)	√		19.4%, *n* = 603
	DASS-21	(46)	√		47.8%, *n* = 1,335
	DASS-21	(47)	√		57.8%, *n* = 816
	DASS-21	(49)	√		48%, *n* = 420
	DASS-21	(50)	√		Mean (SD) = 11.58 (6.98), *n* = 170
	DASS-21	(51)	√		20.5%, *n* = 293
	DASS-21	(52)	√		22.2%, *n* = 338
	DASS-21	(53)	√		8.2%, *n* = 272
	DASS-21	(55)	√		11.1%, *n* = 153
	DASS-21	(56)	√		81.9%, *n* = 751
	DASS-21	(54)	√		47.2%, *n* = 540
	ICC	(82)	√		48.2%, *n* = 678
	IES-R	(57)		√	χ^2^ = 8.34; df = 3; *p* < 0.05. Significantly higher among health professionals compared to general population.
	PSS	(39)	√		88%, *n* = 1,000
	PSS	(72)	√		80.9%, *n* = 444
	Self-questionnaire	(91)		√	χ^2^ = 127.74; df = 6, *n* = 137. Association variable not reported
Other	FCV-19S	(92)	√		Mean (SD) = 18.28 (5.91). Anxiety assessed as fear of COVID-19
Anxiety-related	FCV-19S	(29)	√		57.4%, *n* = 7,381. Assessed as high fear of COVID-19
symptoms	FCV-19S	(53)	√		40%, *n* = 272, assessed as fear of COVID-19
	GHQ-12	(30)		√	39.7% (Males) vs. 60.3% (females), *n* = 317, χ^2^ =12.97. Assessed as gender difference of psychological distress.
	Self-questionnaire	(38)	√		57.5%, *n* = 2,005. Assessed as feeling worried
	Self-questionnaire	(93)	√		87%, *n* = 71. Assessed as fear of getting sick among pregnant women

*AIS, Athens Insomnia Scale; ARS, Anxiety Rating Scale; CAS, Coronavirus Anxiety Scale; CI, Confidence Interval; DASS, Depression, Anxiety and Stress Scale; FCV-19S, Fear of COVID-19 Scale; GAD, Generalized Anxiety Scale; GHQ, General Health Questionnaire; HADS, Hospital Anxiety and Depression Scale; HARS, Hamilton Anxiety Rating Scale; HSCL, Hopkins Symptom Checklist; ICC, Impact of Confinement during Coronavirus; IES, Impact of Event Scale; IES-R, Impact of Event Scale-Revised; ISS, Insomnia severity scale; ISI, Insomnia Severity Index; MBI, Maslach Burnout Inventory; MINI, Mini-International Neuropsychiatric Interview; n, number of participants; OR, Odds Ratio; PHQ, Patient Health Questionnaire; PSS, Perceived Stress Scale; SD, Standard Deviation; SE, Standard Errors*.

### Socio-Demographic Factors Associated With COVID-19 Related Anxiety in Africa

Demographic variables played a significant role in anxiety prevalence. Sex was the most reported demographic variable. Most studies indicated higher levels of anxiety among females compared to males (27, 28, 30, 35, 43, 45–50, 54, 59, 60, 63, 68–70, 72, 73, 75, 81, 83, 94, 95) with the exception of one study which demonstrated an association between anxiety and being male (38). Urban residency (52, 62, 69, 81, 83), living alone (without family) and lower family income or socioeconomic status were also identified as risk factors for anxiety (35, 44, 48, 49, 69). Higher educational status was associated with increased anxiety among healthcare professionals (40, 58, 61, 69) and the general population (42, 49, 62, 83, 92). Anxiety level was higher among other professions compared to healthcare workers (27, 83, 92). Among the various healthcare professions, medical laboratory work was significantly associated with higher anxiety compared to others (AOR = 2.75, 95% CI: 1.78–4.79) (47). Younger age (30, 35, 45, 54, 67, 71, 72, 76, 79, 82, 83, 85), being a widow or single (38, 42, 67, 73), being unemployed (38, 67, 73) and being a student (35, 52, 71) were also significantly associated with COVID-19 related anxiety. Additionally, negative use of religious coping mechanisms was significantly associated with greater anxiety (36, 45).

### Other Associated Factors of COVID-19 Related Anxiety in Africa

Anxiety was highest among those infected with SARS-CoV-2 (OR=9.59, 95% CI: 2.28–40.25) (73) and was significantly associated with having an infected relative or friend (39, 44, 46, 58, 96), being involved in discussions regarding COVID-19 related illness or death (45), fear of contamination (39, 76), fear of death (76) and exposure to individuals with SARS-CoV-2 or who were at risk of having SARS-CoV-2 (15, 43, 58, 74, 95). History of or having an existing mental illness (30, 35, 42, 43, 45, 69) as well as a history of an existing medical condition or chronic disease (46, 49–51, 79, 83, 94, 96) were associated with greater anxiety. Poor knowledge of COVID-19 (48, 49) and preventive practices were associated with increased odds of developing anxiety among pregnant women (41). Similarly, increased COVID-19 related anxiety was significantly associated with being a primigravida (AOR = 3.05, 95% CI: 1.53–6.08), having a gestational age >36 weeks (OR = 5.49, 95% CI: 1.04–28.78) (41), being pregnant (AOR = 4.39, 95% CI: 2.29–12.53) (62) and having a lack of social support while pregnant (AOR = 4.39, 95% CI: 2.29–12.53) (62). Highly protective behavior (AOR = 2.2, 95% CI: 1.5–3.3) and perceived risk behavior (OR = 3.7, 95% CI: 1.5–12.4) predict higher anxiety among the general population (42) but not among healthcare professionals (58). Displacement due to conflict (80), lack of emotional support from family or society (46, 66, 79, 97), and experiencing discrimination or racism (AOR = 5.02, 95% CI: 1.90–13.26) (84) were significantly associated with COVID-19 related anxiety. Reading or watching COVID-19 related news via media or internet sources was associated with increased anxiety (27, 43, 45, 46). Healthcare workers were anxious about their relatives and family members contracting SARS-CoV-2 from them (39, 51, 85, 95). Similarly, long hours working in the hospital (78), fewer years of hospital experience (<3 years) (61), a lack of updated information relating to COVID-19 (74), poor access to personal protective equipment (70, 96), working in a COVID-19 isolation center (54, 60), adult medical-surgical unit (60, 95) or emergency department (58, 60) were significant predictors of higher anxiety. COVID-19 related anxiety was also associated with insomnia among healthcare workers (AOR = 6.38, 95% CI: 4.19–9.73) (54), substance or tobacco use among patients with chronic illness (AOR = 2.27, 95% CI: 1.20–4.30) (79) and alcohol use among the general public (OR 5.50, 95% CI: 2.18–13.87) (75). Among people with disabilities, anxiety-related insomnia was significantly higher among individuals with impaired vision (AOR = 2.8, 95% CI: 1.42–6.35), and hearing (AOR = 10.2, 95% CI: 4.52–35.33) (67).

### Quantitative Synthesis on COVID-19 Related Depression in Africa

#### Overall Prevalence of Depression in Africa During the COVID-19 Pandemic

**Figure 5:**
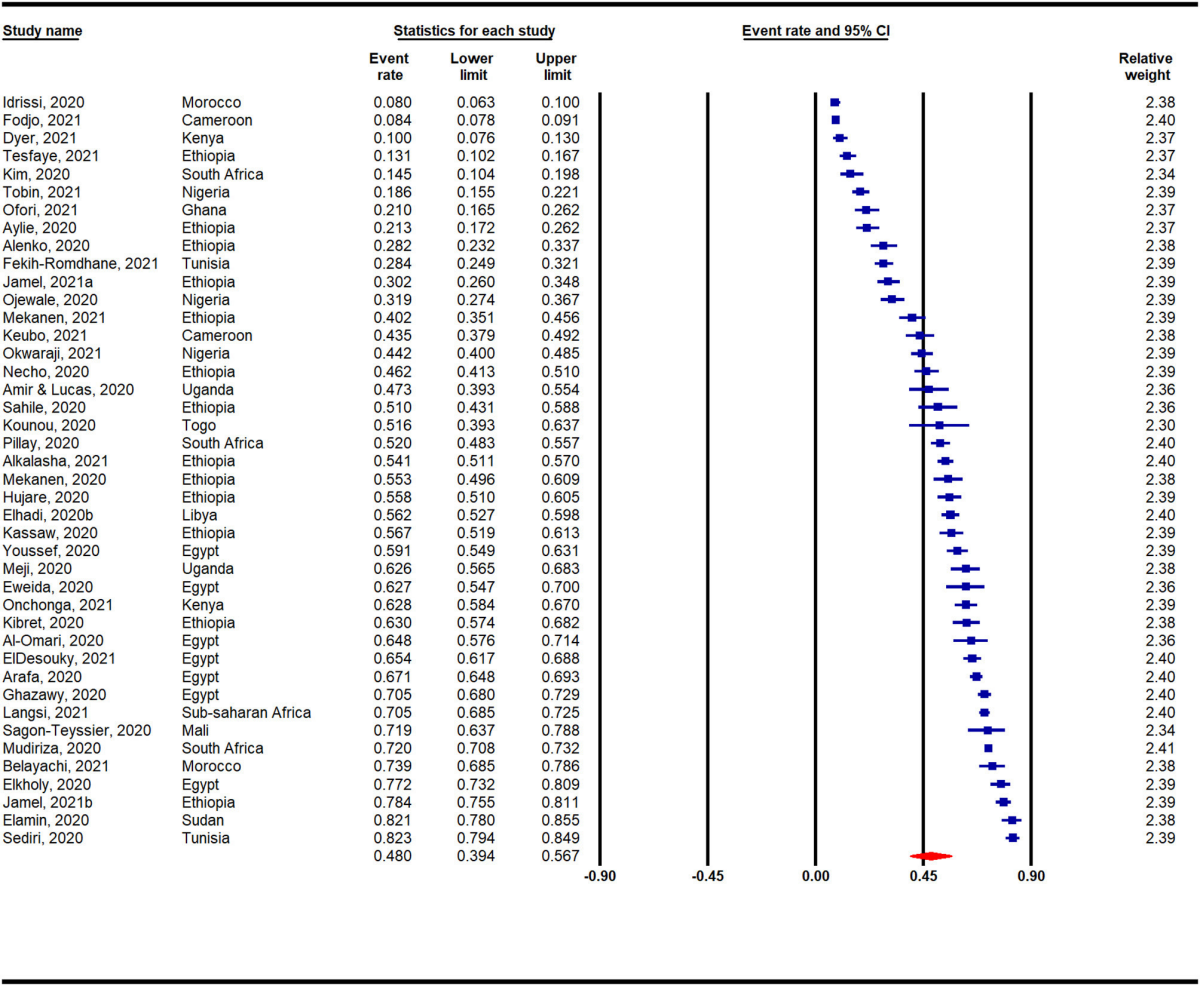
Overall prevalence of depression in Africa during the COVID-19 pandemic.

Figure 5 presents a meta-analysis on the prevalence of depression across Africa during the COVID-19 pandemic. A pooled prevalence rate of 48% (95% CI: 39–57%) was observed for 42 individual studies that surveyed 33,805 participants. Heterogeneity among pooled studies was high (*I*^2^ = 99.45%, *p* < 0.001). The prevalence of depression during the COVID-19 pandemic according to African region is presented in Appendix 6 and Figure 3. A forest plot illustrating further sub-analysis based on the outcome measures utilized to assess depression in the included studies is presented as Appendix 7.

#### Prevalence of Depression in Africa by Countries During the COVID-19 Pandemic

**Figure 6:**
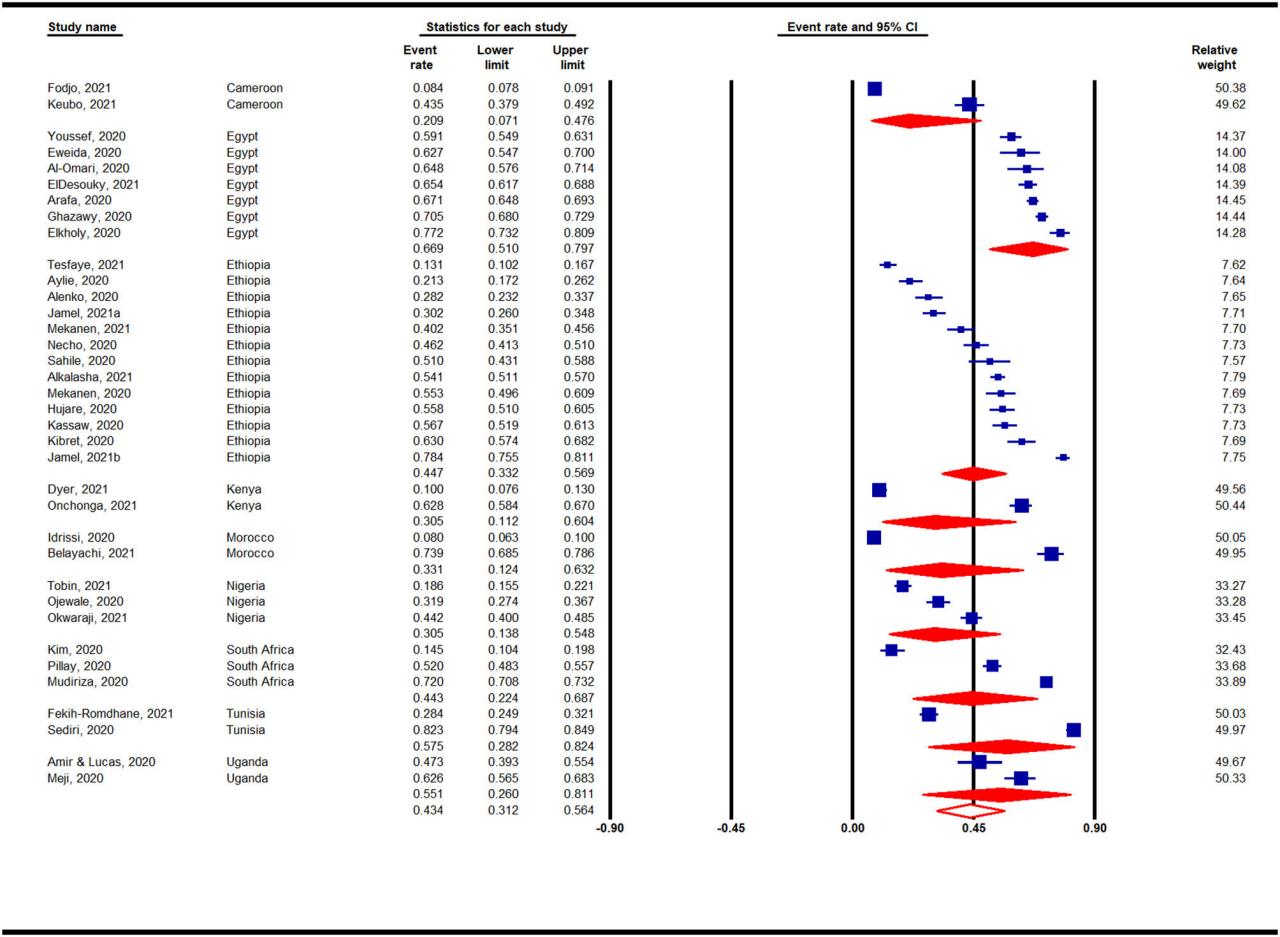
Prevalence of depression in Africa by countries during the COVID-19 pandemic.

Country-based subgroup analyses for depression prevalence indicated that among the nine countries (with ≥ 2 studies) analyzed, the highest prevalence was reported for studies conducted in Egypt (67%, 95% CI: 51–80%, *n* = 7), whereas studies conducted in Nigeria (31%, 95% CI: 14–55%, *n* = 3) and Kenya (31%, 95% CI: 11–60%, *n* = 2) reported the lowest pooled prevalence rates (Figure 6).

#### Prevalence of Depression in Africa by the Period of the COVID-19 Pandemic

Sub-analysis based on the period of the COVID-19 pandemic is presented as Appendix 8. The periods are categorized into Early 2020 (1st January−30th June), Late 2020 (1st July−31st Dec), and Early 2021 (1st January−30th June). Higher prevalence was reported for studies conducted in the Early 2020 (51%, 95% CI: 43–59%, *n* = 6) in comparison with Late 2020 (31%, 95% CI: 14–55%, *n* = 30).

### Narrative Synthesis on COVID-19 Related Depression in Africa

Overall, fifty-nine (*n* = 59) studies reported depression or depressive symptoms (Table 2). The prevalence of depression ranged from 8% (*n* = 846) among the general population in Morocco (81) to 82.3% (*n* = 751) among women in Tunisia (56) (Table 2). DASS-21 (*n* = 17) and PHQ (*n* = 17) were the most commonly used measures of depression, with proportions ranging from 21.1 to 82.3% and 8.2 to 82%, respectively. Depression scores were significantly correlated with overall anxiety (14, 35, 40, 50) and sleeplessness (14, 54). Overall depression was significantly associated with symptoms of mania (AOR = 4.3, 95% CI: 1.71–11.02) (84) and Fear of Covid scores (*r* = 0.5, *p* < 0.001) (77). Depression-related symptoms included reports of boredom (94), poor mood (82), loneliness (91), loss of interest (101), total emotional exhaustion (77, 78) and suicidal ideation or behavior (71, 84) (Table 2).

**Table 2:** Depression-related COVID-19 effect in Africa.

**Outcome**	**Measure**	**References**	**Effect of COVID-19 on the outcome**		**Effect size/comment**
			**Overall reported**	**No overall reported**	
Overall depression	BDS	(81)	√		8%, *n* = 827
prevalence	BDI-2	(64)	√		44.2%, *n* = 520
	CES-D 10	(31)	√		72%, *n* = 5,693
	DASS-21	(43)	√		64.8%, *n* = 182
	DASS-21	(37)	√		Mean (SE) = 11.63 (1.04); *n* = 83. Assessed for three African countries (South Africa, Egypt, Uganda)
	DASS-21	(27)	√		67.1%, *n* = 1,629
	DASS	(44)	√		21.3%, *n* = 314
	DASS-21	(35)	√		Mean (SD) = 9.74(9.70), *n* = 264 Algeria; 10.62(9.68), *n* = 293 Egypt; 5.53(7.49), *n* = 435 Nigeria
	DASS-21	(45)	√		28.3%, *n* = 603
	DASS-21	(46)	√		70.5%, *n* = 1,335
	DASS-21	(47)	√		78.4%, *n* = 816
	DASS-21	(48)	√		44.4%, *n* = 420. The outcome was assessed as general mental health crises, comprising of depression, anxiety, and stress.
	DASS-21	(49)	√		56.6%, *n* = 420
	DASS-21	(50)	√		Mean (SD) = 12.54 (6.72), *n* = 170
	DASS-21	(51)	√		55.3%, *n* = 293
	DASS-21	(52)	√		40.2%, *n* = 338
	DASS-21	(53)	√		21.1%, *n* = 272
	DASS-21	(54)	√		59.1%, *n* = 540
	DASS-21	(55)	√		51%, n = 153
	DASS-21	(56)	√		82.3%, *n* = 751
	GAD-7	(96)	√		63%, *n* = 305
	GHQ-12	(14)	√		Mean (SD) = 12.54(4.58), *n* = 182
	GHQ	(95)	√		62.7%, *n* = 150
	GHQ-28	(98)	√		14.5%, *n* = 221
	HADS	(77)	√		Mean (SD)=9.1(3.2), n = 320
	HADS	(28)	√		73.9%, *n* = 287
	HADS	(78)	√		Mean (SD) = 12.39 (2.95), *n* = 154
	HADS	(80)	√		56.3%, *n* = 745
	HADS	(79)	√		55.7%, *n* = 423. Among patients with chronic medical conditions
	HADS	(76)	√		43.5%, *n* = 292
	HADS	(32)	√		31.9%, *n* = 386
	HSCL	(34)	√		Mean (SD) = 0.96 (1.09), *n* = 626 Congo, 1.16 (1.14), *n* = 174 Rwanda and 0.53 (0.61), *n* = 242 Togo
	MINI	(84)	√		13.1%, *n* = 420
	PHQ	(40)	√		28.1%, *n* = 277
	PHQ	(65)		√	χ^2^ = 1.94, df = 4, *P* > 0.05. The assessed difference among gender.
	PHQ-9	(29)	√		8.4%, *n* = 7,381
	PHQ-9	(75)	√		18.6%, *n* = 543
	PHQ-9	(99)	√		47%, *n* = 146
	PHQ-9	(33)	√		10%, *n* = 479 among young adults with HIV (10–24 years)
	PHQ-9	(59)	√		65.3%, *n* = 687
	PHQ-9	(58)	√		82%, *n* = 396
	PHQ-9	(71)	√		Mean (SD) = 9.7 (6.3), *n* = 2,430
	PHQ-9	(72)	√		77.2%, *n* = 457
	PHQ-9	(70)	√		71.9%, *n* = 135
	PHQ-9	(60)	√		30.2%, *n* = 417
	PHQ-9	(63)	√		51.6%, *n* = 62
	PHQ-9	(67)	√		46.2%, *n* = 403, people with disability
	PHQ-9	(68)	√		62.8%, *n* = 476
	PHQ-9	(69)	√		54.1%, *n* = 1,080
	PHQ-9	(57)		√	χ^2^ = 14.26; df = 4, *n* = 884. Higher among health professionals compared to the general population.
	Self-Questionnaire	(91)		√	χ^2^ = 89.24; df = 6, *n* = 137. Association variables not reported
	Self-Questionnaire	(38)	√		70.5%, *n* = 2,005
	Self-Questionnaire	(86)	√		62.7%, *n* = 254
	Self-Questionnaire	(100)	√		52%, *n* = 692
	Self-Questionnaire	(89)		√	Mean (SD) for males = 8.59 (3.25) and females = 10.7 (4.21), *t* = 2.24, *n* = 69.
Other	BPS	(94)	√		43.2%, *n* = 811. Assessed as feeling boredom. Increased compared to before COVID-19
depressive-related	ICC	(82)	√		46.6%, *n* = 678, feeling of a bad mood
symptoms	MBI	(77)	√		Mean (SD) =24.5(9.4). Assessed as total emotional exhaustion
	MBI	(78)	√		Mean (SD) = 11.2 (3.81). Assessed as total emotional exhaustion
	MINI	(84)	√		5.5%%, *n* = 420. Assessed as suicide behavior
	PHQ-9	(71)	√		22.7%, *n* = 3,500. Assessed as suicidal ideation.
	Self-questionnaire	(91)		√	χ^2^ = 88.38; df = 6. Assessed as feeling loneliness
	Self-questionnaire	(101)	√		50.3%, *n* = 900. Assessed as difficulty in work and loss of interest.
	Self-Questionnaire	(102)	√		94%, *n* = 415. Assessed as feeling depressed/hopeless/fear of COVID-19

*BDI, Beck Depression Inventory; BPS, Boredom Proneness Scale; CES-D, Center for Epidemiological Studies on Depression; CI, Confidence Interval; DASS, Depression, Anxiety and Stress Scale; FCV-19S, Fear of COVID-19 Scale; GHQ, General Health Questionnaire; HADS, Hospital Anxiety and Depression Scale; HSCL, Hopkins Symptom Checklist; ICC, Impact of Confinement during Coronavirus; IES, Impact of Event Scale; IES-R, Impact of Event Scale-Revised; ISI, Insomnia Severity Index; MBI, Maslach Burnout Inventory; MINI, Mini-International Neuropsychiatric Interview; n, number of participants; OR, Odds Ratio; PHQ, Patient Health Questionnaire; SD, Standard Deviation; SE, Standard Errors*.

### Socio-Demographic Factors Associated With COVID-19 Related Depression in Africa

Among the demographic characteristics of the respondents, older age (≥65 years) was associated with a decrease in depression or depressive symptoms (34, 80) (35, 45, 59, 81, 94), while another study reported greater depression among middle-aged people (45–65 years) (58). The prevalence of depression was significantly higher among females than males (27, 28, 31, 34, 35, 43–50, 59, 60, 63, 72, 79, 81, 94, 99). Marital status (i.e., being single, widowed, divorced, or separated) (59, 67, 79), living alone without family or having a lack of emotional support from family and society (71, 97) (45, 46), family size ≥ 3 (48, 49) and living in an urban area (31, 32, 69) were found to significantly increase depression, with the exception of two studies (47, 60) which indicated that married people were more than three times as likely to experience depression (*p* < 0.05). Higher educational level and professional qualification were found to significantly increase depression (29, 31, 49, 59, 60, 69) with the exception of one study, which indicated higher depressive symptoms among non-educated people with disability (AOR = 2.12, 95% CI: 1.12–5.90) (67). Unemployment (AOR = 2.1, 95% CI: 1.32–5.11) (67) and low socioeconomic status significantly influenced the prevalence of depressive symptoms (35, 48, 49, 57, 69). Among healthcare professions, medical lab workers (AOR = 4.69, 95% CI: 2.81–9.17) were more likely to experience depression (47), followed by nurses (47, 63). Religion also contributed to the prevalence of depression, with negative use of religious coping mechanisms being associated with greater depression (*r* = 0.135) (45).

### Other Associated Factors of COVID-19 Related Depression in Africa

The prevalence of depressive symptoms was significantly higher among healthcare professionals compared to the general population (χ^2^ = 14.26, *p* < 0.01) (57). In the healthcare sector, depression prevalence was associated with working in the emergency department (58, 60, 80), fewer years of experience (60), working in a surgical unit (80), working in a COVID-19 isolation center in comparison to other units (AOR = 2.14, 95% CI: 1.05–4.39) (47) and in fever hospitals compared to designated COVID-19 quarantine hospitals (OR = 1.52, 95% CI: 1.11–2.09) (72). Similarly, nurses who received negative feedback from their families (AOR = 2.19, 95% CI: 1.27–3.79) and a lack of COVID-19 management guidelines (AOR = 2.26, 95% CI: 1.21–4.21) were more likely to experience depression (51). Depression was associated with a history of other medical conditions or chronic disease (32, 44–46, 49, 63, 94), flu-like symptoms (29), fear of death (76), the recent death of a loved one (84), quarantine or home stay (29, 43, 44) and poor social support among students (35, 44, 46) and chronic medical patients (79). Reading or watching COVID-19 related news via media or internet sources was associated with increased depression (43, 45, 97). A history of or existing mental illness (35, 43, 51), the current use of medication (75), perceived COVID-19 risk (98), and exposure to individuals with SARS-CoV-2 at risk of having contracted SARS-CoV-2 (43) or having a family member with COVID-19 positive test results (52) significantly increased the odds of COVID-19 related depression. Prevalence of depression was significantly associated with insomnia (AOR = 7.58, 95% CI: 4.91–11.68) among healthcare workers (54), and alcohol use among the general population (OR 4.27, 95% CI: 1.56–12.04, *p* < 0.01) (75). Prevalence of depression was also associated with conflict-affected regions (71). Stigma significantly affected symptoms of depression (34, 80).

## Discussion

This study aimed to systematically review the literature on the prevalence and risk factors associated with anxiety and depression among the general populace in Africa during the COVID-19 pandemic. To our knowledge, this review is the first most comprehensive multidimensional synthesis of COVID-19 associated depression and anxiety prevalence across the African continent. Although new data is rapidly emerging, the prevalence reported in African countries has largely been overlooked in similar reviews encompassing the global population (16, 21), while a recent African-based systematic review in this area of research focused only on healthcare workers (18) or included only limited number of studies (103). Although no causal inference can be made given the interplay of other related factors (i.e., lockdowns, misinformation, civil unrest, etc.), our evidence suggests the COVID-19 pandemic had a substantial impact on anxiety and depression among the general populace in Africa. The meta-analysis results indicated a high overall prevalence of anxiety (47%). Regionally, the highest prevalence of anxiety was seen in Eastern Africa (49%), with the lowest in the Middle African region (42%). Furthermore, the overall prevalence of depression in Africa (48%) was comparable to the prevalence of anxiety. Northern and Middle African regions had the highest (61%) and lowest (21%) prevalence rates of depression in the continent, respectively. Commonly reported risk factors for anxiety and depression were sex (i.e., female) and a history of chronic medical conditions. Age was a risk factor for both psychological symptoms, with lower and higher (≥60 year) ages relating to greater levels of anxiety and depression, respectively.

To contextualize the present findings in the existing literature, a previous review on the global prevalence of anxiety disorders reported Africa to have a lower prevalence (5.3%) compared to Europe (10.4%) and the United States (0.7–16.2%) before the emergence of COVID-19 (104, 105). Similarly, depression was found to be higher in Southeast Asian and Western Pacific regions (106). However, with the recent COVID-19 pandemic, the global prevalence of mental health problems increased exponentially. The pooled prevalence of anxiety in this review was higher than that reported in China (22–28%) (13, 107, 108), and South Asian countries (41.3%) (109). The prevalence of depression was also reported to be higher in Africa compared to the United States (5.1–24.6%) (105) and China (22%) among healthcare workers. A collation of studies published at the early stage of the COVID-19 pandemic (between January 2020-April 2021), reported wider ranges of prevalence rates of anxiety (9.5–73.3%) and depression (12.5–71.9%) among healthcare workers in Africa (18). Our meta-analytic findings on the overall prevalence of anxiety and depression are slightly higher in comparison with an earlier systematic review and meta-analysis (between February 2020-February 2021) that reported 37% and 45% prevalence rates of anxiety and depression, respectively (103). Apart from the fact that our search for included studies extends to the end of September 2021, the inclusion of fewer studies by Chen et al. (*n* = 28), might have resulted in the discrepant findings.

In the narrative synthesis, we report a wide-spread prevalence of COVID-19 related-anxiety (11.3–88.5%) and depression (8.0–82.3%) across the included studies. Pooled prevalence rates reported for anxiety (47%) and depression (48%) were roughly equivalent, suggesting that these symptoms may occur in tandem rather than independently of one another. It should be noted that the largest proportion of studies reported in this review also represented countries with the largest GDP and more developed healthcare infrastructure in Africa (i.e., Nigeria, Egypt, Ethiopia). This suggests that in most other African countries with comparatively lower GDP and less developed healthcare infrastructure, the prevalence of anxiety and depression is likely to be either understudied, underreported or presumably worse. Libya was the only exception, with a relatively lower prevalence of anxiety (28%) in comparison to most other countries in Africa. Although on-going geopolitical events (e.g., civil war, change in political leadership, etc.) possibly overshadow the effect of the pandemic in terms of an attributable cause of anxiety (71), the number of studies was also limited (i.e., *n* = 2) (73, 80), suggesting that this subgroup may be comparatively underpowered. More large-scale survey studies need to be conducted in impoverished African countries to produce a broader and more comprehensive picture of COVID-19 related anxiety and depression prevalence.

The concomitant effects of socioeconomic, political, and managerial challenges are also factors attributed to the higher prevalence of anxiety and depression. Many countries in the African continent face healthcare challenges owing to a limited number of healthcare workers, insufficient budgetary allocation to improve healthcare infrastructure, and poor leadership coupled with inter-professional conflict among healthcare workers, ultimately making the health system less effective in handling the COVID-19 pandemic (9, 12). Furthermore, other indirect factors such as endemic poverty, large disparities between the wealthy and the impoverished, geopolitical instability and insecurity (e.g., Boko Haram, the Islamic State's West Africa Province, banditry, and communal clashes), high rates of unemployment, and an unequal or ineffective social support system further exacerbate the burden associated with the pandemic response in comparison to other continents (10). Together, these factors make it difficult for the general population to cope with and adhere to broad mandates and preventative measures such as lockdowns, social distancing, or quarantines as many individuals and families struggle economically. The overall impact translates to a reduced quality of life and a general decline in mental health (e.g., anxiety and depression) (11).

As wealthy countries progress expeditiously toward general immunity through large scale, fully subsidized, and newly mandated vaccination programs (110), many developing countries, and indeed entire continents, are bracing to prepare for potentially endemic COVID-19. The hesitancy of G7 countries to pledge support for the provision of initial vaccine doses to other developing countries while concomitantly stockpiling booster vaccines (111), will inevitably widen the global divide between those who are vaccinated and unvaccinated, a distinction now being referred to “the jabs and the jab-nots” (112). Normalization of community mask-wearing, particularly in the rural areas of developing countries, not only require the free distribution of masks but also multifaceted promotion strategies (e.g., text reminders, signage, advocacy by local religious leaders, etc.) for eliciting changes in social norms as the driver of sustained, community-wide behavior change (113). Policies on the adoption of vaccine passports, while meant to incentivize vaccination among the general public, are replete with ethical and legal concerns (114). Within Africa, the public perception of these impending circumstances may potentially exacerbate symptoms of COVID-19 related anxiety and depression (29, 53, 92), in addition to the possibility of novel viral strains that are likely to emerge in subsequent waves (5). The anticipatory distress of potentially contracting the virus (30, 38, 93) among the overall population (encompassing frontline health care workers) (87) is contributing to what we see as an emerging mental health crisis throughout the African continent (36).

Our study has several strengths. Firstly, we conducted a systematic review and meta-analyses on the overall prevalence of anxiety and depression in the African continent during the COVID-19 pandemic and various sub-analyses based on the African countries, regions, outcome measures and study period. Secondly, our literature search was robust, encompassing a search theme that included all the countries in Africa, and we searched the “African Journal Online” database to ensure wider coverage of published studies in the research area. Finally, we adopted an appropriate methodological quality checklist (the Agency for Healthcare Research and Quality) in the study. However, this review has some limitations that should be considered when interpreting the reported findings. Firstly, our search was limited to articles published in the online academic journals. Therefore, locally published articles in African-based non-indexed journals might have not been identified during the literature search process. Secondly, due to high heterogeneity reported in the study, care should be taken when interpreting the meta-analyses. Finally, we excluded conference abstracts and non-English language studies from the review, which may limit the external validity of the study.

## Conclusions

Understanding the current prevalence of anxiety and depression in Africa is important for the allocation of resources dedicated to mental healthcare providers and related education programs. In the coming year, broader access to vaccines and masks, and the societal pressure to either adopt or avoid these preventative strategies are also potential sources of anxiety and depression. This may be ameliorated through the robust implementation of COVID-19-related education programs and subsequent provision of mental healthcare.

## Data Availability Statement

The original contributions presented in the study are included in the article/Supplementary Material, further inquiries can be directed to the corresponding author/s.

## Author Contributions

UB: accessed and verified data, conceptualization, article electronic search, article screening, meta-analysis, and manuscript drafting. PK and AC: conceptualization, manuscript drafting, and project supervision. MC: accessed and verified data, conceptualization, narrative synthesis, meta-analysis, and manuscript drafting. DS: accessed and verified data, conceptualization, manual search narrative synthesis, meta-analysis, and manuscript drafting. TM: quality rating and manuscript drafting. JP: article electronic search. AM and FM: data extraction. HJ, IB, and AG: manual search, and manuscript drafting. MA: quality rating. MK: narrative synthesis. SS and AL: manuscript drafting and data curation. SW: conceptualization, article electronic search, articles screening, manuscript drafting, and project supervision. All authors had full access to the data and are in general agreement with the decision to submit the manuscript for publication.

## Funding

The work of UB and AC was supported by the Hong Kong Special Administrative Region Government and InnoHK.

## Conflict of Interest

UB and AC were employed by the Centre for Eye and Vision Research (CEVR) Limited. The remaining authors declare that the research was conducted in the absence of any commercial or financial relationships that could be construed as a potential conflict of interest.

## Publisher's Note

All claims expressed in this article are solely those of the authors and do not necessarily represent those of their affiliated organizations, or those of the publisher, the editors and the reviewers. Any product that may be evaluated in this article, or claim that may be made by its manufacturer, is not guaranteed or endorsed by the publisher.
