# Supplementary material for: Prevalence of Anxiety and Depression Among the General Population in Africa During the COVID-19 Pandemic: A Systematic Review and Meta-Analysis
Source: Front Public Health. 2022 May 17;10:814981. doi: 10.3389/fpubh.2022.814981 (PMC9152218; doi:10.3389/fpubh.2022.814981)
Supplement: Supplementary file 1 [file Data_Sheet_1.docx]

**SUPPLEMENTARY MATERIALS**

Appendix 1a: Literature search terms

| Search themes  *COVID-19 Anxiety & Depression Africa* | | | |
| --- | --- | --- | --- |
| *Relating terms* | “COVID-19”  “SARS-CoV-2” “Corona virus”  “Corona virus disease”  “Corona virus Pandemic” | “Anxiety”  “Depression”  “Depressive symptoms”  “Psychological distress” | “Africa”  “Sub-Saharan Africa”  “Algeria”  “Angola”  “Benin”  “Botswana”  “Burkina Faso”  “Burundi”  “Cameroon”  “Cape Verde”  “The Central Africa Republic”  “Chad”  “Comoros”  “The Democratic Republic of Congo”  “Republic of Congo”  “Cote d’Ivoire”  “Djibouti”  “Egypt”  “Equatorial Guinea”  “Eritrea”  “Ethiopia”  “Gabon”  “The Gambia”  “Ghana”  “Guinea”  “Guinea-Bissau”  “Kenya”  “Lesotho”  “Liberia”  “Libya”  “Madagascar”  “Malawi”  “Mali”  “Mauritius”  “Morocco”  “Mozambique”  “Namibia”  “Niger”  “Nigeria”  “Rwanda”  “Sao Tome and Principe”  “Senegal”  “Seychelles”  “Sierra Leone”  “Somalia”  “South Africa”  “Sudan (North)”  “South Sudan”  “Swaziland”  “Tanzania”  “Togo”  “Tunisia”  “Uganda”  “Zambia”  “Zimbabwe” |

Appendix 1bi: CINAHL database search strategy (Search date: 30^th^ September 2021)

| Search themes | Search terms |
| --- | --- |
| #1 (*COVID-19)* | COVID-19 OR SARS-CoV-2 OR Corona virus OR Corona virus disease OR Corona virus Pandemic |
| #2 (*Anxiety & Depression)* | Anxiety OR Depression OR Depressive symptoms OR Psychological distress |
| #3 (*Africa)* | Africa OR Sub-Saharan Africa OR Algeria OR Angola OR Benin OR Botswana OR Burkina Faso OR Burundi OR Cameroon OR Cape Verde OR The Central Africa Republic OR Chad OR Comoros OR The Democratic Republic of Congo OR Republic of Congo OR Cote d’Ivoire OR Djibouti OR Egypt OR Equatorial Guinea OR Eritrea OR Ethiopia OR Gabon OR The Gambia OR Ghana OR Guinea OR Guinea-Bissau OR Kenya OR Lesotho OR Liberia OR Libya OR Madagascar OR Malawi OR Mali OR Mauritius OR Morocco OR Mozambique OR Namibia OR Niger OR Nigeria OR Rwanda OR Sao Tome and Principe OR Senegal OR Seychelles OR Sierra Leone OR Somalia OR South Africa OR Sudan (North) OR South Sudan OR Swaziland OR Tanzania OR Togo OR Tunisia OR Uganda OR Zambia OR Zimbabwe |
| #4 | #1AND#2AND#3 |

Appendix 1bii: PubMed database search strategy (Search date: 30^th^ September 2021)

| Search themes | Search terms |
| --- | --- |
| #1 (*COVID-19)* | COVID-19 OR SARS-CoV-2 OR Corona virus OR Corona virus disease OR Corona virus Pandemic |
| #2 (*Anxiety & Depression)* | Anxiety OR Depression OR Depressive symptoms OR Psychological distress |
| #3 (*Africa)* | Africa OR Sub-Saharan Africa OR Algeria OR Angola OR Benin OR Botswana OR Burkina Faso OR Burundi OR Cameroon OR Cape Verde OR The Central Africa Republic OR Chad OR Comoros OR The Democratic Republic of Congo OR Republic of Congo OR Cote d’Ivoire OR Djibouti OR Egypt OR Equatorial Guinea OR Eritrea OR Ethiopia OR Gabon OR The Gambia OR Ghana OR Guinea OR Guinea-Bissau OR Kenya OR Lesotho OR Liberia OR Libya OR Madagascar OR Malawi OR Mali OR Mauritius OR Morocco OR Mozambique OR Namibia OR Niger OR Nigeria OR Rwanda OR Sao Tome and Principe OR Senegal OR Seychelles OR Sierra Leone OR Somalia OR South Africa OR Sudan (North) OR South Sudan OR Swaziland OR Tanzania OR Togo OR Tunisia OR Uganda OR Zambia OR Zimbabwe |
| #4 | #1AND#2AND#3 |

Appendix 1biii Scopus database search strategy (Search date: 30^th^ September 2021)

| Search themes | Search terms |
| --- | --- |
| #1 (*COVID-19)* | COVID-19 OR SARS-CoV-2 OR Corona virus OR Corona virus disease OR Corona virus Pandemic |
| #2 (*Anxiety & Depression)* | Anxiety OR Depression OR Depressive symptoms OR Psychological distress |
| #3 (*Africa)* | Africa OR Sub-Saharan Africa OR Algeria OR Angola OR Benin OR Botswana OR Burkina Faso OR Burundi OR Cameroon OR Cape Verde OR The Central Africa Republic OR Chad OR Comoros OR The Democratic Republic of Congo OR Republic of Congo OR Cote d’Ivoire OR Djibouti OR Egypt OR Equatorial Guinea OR Eritrea OR Ethiopia OR Gabon OR The Gambia OR Ghana OR Guinea OR Guinea-Bissau OR Kenya OR Lesotho OR Liberia OR Libya OR Madagascar OR Malawi OR Mali OR Mauritius OR Morocco OR Mozambique OR Namibia OR Niger OR Nigeria OR Rwanda OR Sao Tome and Principe OR Senegal OR Seychelles OR Sierra Leone OR Somalia OR South Africa OR Sudan (North) OR South Sudan OR Swaziland OR Tanzania OR Togo OR Tunisia OR Uganda OR Zambia OR Zimbabwe |
| #4 | #1AND#2AND#3 |

Appendix 1biv: Web of Science database search strategy (Search date: 30^th^ September 2021)

| Search themes | Search terms |
| --- | --- |
| #1 (*COVID-19)* | COVID-19 OR SARS-CoV-2 OR Corona virus OR Corona virus disease OR Corona virus Pandemic |
| #2 (*Anxiety & Depression)* | Anxiety OR Depression OR Depressive symptoms OR Psychological distress |
| #3 (*Africa)* | Africa OR Sub-Saharan Africa OR Algeria OR Angola OR Benin OR Botswana OR Burkina Faso OR Burundi OR Cameroon OR Cape Verde OR The Central Africa Republic OR Chad OR Comoros OR The Democratic Republic of Congo OR Republic of Congo OR Cote d’Ivoire OR Djibouti OR Egypt OR Equatorial Guinea OR Eritrea OR Ethiopia OR Gabon OR The Gambia OR Ghana OR Guinea OR Guinea-Bissau OR Kenya OR Lesotho OR Liberia OR Libya OR Madagascar OR Malawi OR Mali OR Mauritius OR Morocco OR Mozambique OR Namibia OR Niger OR Nigeria OR Rwanda OR Sao Tome and Principe OR Senegal OR Seychelles OR Sierra Leone OR Somalia OR South Africa OR Sudan (North) OR South Sudan OR Swaziland OR Tanzania OR Togo OR Tunisia OR Uganda OR Zambia OR Zimbabwe |
| #4 | #1AND#2AND#3 |

**Appendix 2: The Agency for Healthcare Research and Quality (AHRQ) methodological checklist for observational studies reporting the quality of the included studies**

| **Study reference** | **1** | **2** | **3** | **4** | **5** | **6** | **7** | **8** | **9** | **10** | **Total score** |
| --- | --- | --- | --- | --- | --- | --- | --- | --- | --- | --- | --- |
| Abdelghani et al., (2020) | Y | N | N | Y | Y | Y | Y | Y | Y | Y | 8 |
| Afolabi (2020) | Y | N | N | N | Y | Y | Y | Y | N | N | 5 |
| Agberotimi et al., (2020) | Y | Y | N | Y | Y | Y | Y | Y | N | N | 7 |
| Akorede et al., (2021) | Y | N | N | N | Y | Y | Y | Y | N | N | 5 |
| AlKalasha & Kasemy (2020) | Y | N | N | Y | Y | Y | Y | Y | N | Y | 7 |
| Al Omari et al., (2020) | Y | Y | N | Y | Y | Y | Y | Y | Y | Y | 9 |
| Alenko et al., (2021) | Y | Y | Y | Y | Y | Y | Y | Y | Y | Y | 10 |
| Aluh et al., (2021) | Y | Y | N | Y | Y | Y | Y | Y | N | N | 7 |
| Alzueta et al., (2021) | Y | Y | Y | Y | Y | Y | Y | Y | Y | N | 9 |
| Amir & Lucas (2021) | Y | Y | Y | Y | Y | Y | N | Y | N | Y | 8 |
| Anikwe et al., (2021) | Y | Y | Y | Y | Y | Y | Y | Y | N | Y | 9 |
| Arafa et al., (2021a) | Y | Y | Y | Y | Y | Y | Y | Y | Y | Y | 10 |
| Arafa et al., (2021b) | Y | Y | Y | Y | Y | Y | Y | Y | Y | Y | 10 |
| Aylie et al., (2020) | Y | Y | Y | Y | Y | Y | Y | Y | Y | Y | 10 |
| Belayachi et al., (2021) | Y | N | N | Y | Y | Y | Y | Y | N | N | 6 |
| Birhanu et al., (2021) | Y | N | N | Y | Y | Y | Y | Y | N | Y | 7 |
| Boateng et al., (2021) | Y | Y | N | Y | Y | Y | Y | Y | N | Y | 8 |
| Cénat et al., (2021a) | Y | N | N | Y | Y | Y | Y | Y | Y | N | 7 |
| Cénat et al., (2021b) | Y | N | N | Y | Y | Y | Y | Y | Y | N | 7 |
| Dyer et al., (2021) | Y | Y | N | Y | Y | Y | Y | Y | N | N | 7 |
| Eisenbeck et al., (2021) | Y | N | Y | Y | Y | Y | Y | Y | N | N | 7 |
| Ejeh et al., (2021) | Y | N | N | Y | Y | Y | Y | Y | Y | Y | 8 |
| El Desouky et al., (2021) | Y | N | N | Y | Y | Y | Y | Y | Y | N | 7 |
| Elamin et al., (2020) | Y | N | N | Y | Y | Y | Y | Y | N | N | 6 |
| Elhadi et al., (2020a) | Y | Y | Y | Y | Y | Y | Y | Y | N | Y | 9 |
| Elhadi et al., (2020b) | Y | Y | Y | Y | Y | Y | Y | N | N | Y | 8 |
| Elhadi et al., (2021) | Y | Y | Y | Y | Y | Y | Y | Y | N | Y | 9 |
| Elkholy et al., (2020) | Y | Y | Y | Y | Y | Y | N | Y | Y | Y | 9 |
| El-Zoghby et al., (2020) | Y | Y | Y | Y | Y | Y | Y | Y | Y | N | 9 |
| Eweida et al., (2020) | Y | Y | Y | Y | Y | Y | Y | Y | Y | N | 9 |
| Fekih-Romdhane & Cheour (2021) | Y | Y | N | Y | Y | Y | Y | Y | Y | Y | 9 |
| Fodjo et al., (2021) | Y | N | N | Y | Y | Y | Y | N | Y | Y | 7 |
| Ghazawy et al., (2020) | Y | N | N | Y | Y | Y | N | Y | Y | N | 6 |
| Hajure et al., (2020) | Y | Y | Y | Y | Y | Y | Y | Y | Y | Y | 10 |
| Idowu et al., (2020) | Y | N | N | Y | Y | Y | Y | Y | Y | Y | 8 |
| Idrissi et al., (2020) | Y | N | N | Y | Y | Y | N | Y | Y | N | 6 |
| Jemal et al., (2021a) | Y | Y | Y | Y | Y | Y | Y | Y | Y | Y | 10 |
| Jemal et al. (2021b) | Y | Y | Y | Y | Y | Y | Y | Y | Y | Y | 10 |
| Kassawa & Ali (2020) | Y | Y | Y | Y | Y | Y | N | Y | Y | N | 8 |
| Kassawa & Pandey (2020) | Y | Y | Y | Y | Y | Y | N | Y | Y | Y | 9 |
| Kassaw & Pandey (2021) | Y | Y | Y | Y | Y | Y | N | Y | Y | N | 8 |
| Kassaw (2020) | Y | Y | Y | Y | Y | Y | N | Y | Y | N | 8 |
| Keubo et al., (2021) | Y | Y | N | Y | Y | Y | N | Y | N | N | 6 |
| Khalaf et al., (2020) | Y | Y | N | Y | Y | Y | N | Y | N | N | 6 |
| Kibret et al., (2020) | Y | Y | N | Y | Y | Y | Y | Y | Y | Y | 9 |
| Kim et al., (2020) | Y | Y | N | Y | Y | Y | Y | Y | Y | N | 8 |
| Kounou et al., (2020) | Y | N | N | Y | Y | Y | N | Y | N | N | 5 |
| Lamptey (2020) | Y | N | N | Y | Y | Y | N | Y | Y | Y | 7 |
| Langsi et al., (2021) | Y | Y | Y | Y | Y | Y | Y | Y | Y | N | 9 |
| Madani et al., (2020) | Y | N | N | Y | Y | Y | Y | Y | N | N | 6 |
| Meji & Dennison (2020) | Y | Y | N | Y | Y | Y | N | Y | N | N | 6 |
| Mekonen et al., (2021) | Y | Y | Y | Y | Y | Y | Y | N | Y | Y | 9 |
| Mekonen et al., (2020) | Y | Y | Y | Y | Y | Y | Y | N | Y | Y | 9 |
| Moyer et al., (2021) | Y | N | N | Y | Y | Y | N | Y | N | N | 5 |
| Msherghi et al., (2021) | Y | N | N | Y | Y | Y | Y | Y | Y | N | 7 |
| Mudenda et al., (2021) | Y | Y | Y | Y | Y | Y | N | N | Y | Y | 8 |
| Mudiriza & De Lannoy (2020) | Y | Y | Y | Y | Y | Y | Y | N | Y | Y | 9 |
| Necho et al., (2020) | Y | Y | Y | Y | Y | Y | Y | Y | Y | Y | 10 |
| Odikpo et al., (2021) | Y | N | N | Y | Y | Y | N | Y | N | Y | 6 |
| Ofori et al., (2021) | Y | Y | Y | Y | Y | Y | Y | Y | N | Y | 9 |
| Ojewale (2020) | Y | N | N | N | Y | Y | Y | Y | Y | N | 6 |
| Okwaraji & Onyebueke (2021) | Y | Y | N | Y | Y | Y | Y | Y | N | Y | 8 |
| Olaseni et al., (2020) | Y | Y | N | Y | Y | Y | Y | Y | N | N | 7 |
| Olashore et al., (2021b) | Y | Y | Y | Y | Y | Y | Y | Y | Y | Y | 10 |
| Onchonga et al., (2021) | Y | Y | N | N | Y | Y | Y | Y | Y | N | 7 |
| Pillay et al., (2020) | Y | Y | N | Y | Y | Y | Y | Y | N | Y | 8 |
| Rakhmanov & Dane (2020) | Y | Y | Y | Y | Y | Y | N | Y | N | N | 7 |
| Rakhmanov et al., (2020a) | Y | Y | Y | Y | Y | Y | Y | Y | N | N | 8 |
| Rakhmanov et al., (2020b) | Y | N | N | Y | Y | Y | Y | Y | N | N | 6 |
| Sagaon-Teyssier et al., (2020) | Y | Y | N | Y | Y | Y | N | N | Y | Y | 7 |
| Sahile et al., (2020) | Y | Y | Y | Y | Y | Y | N | Y | N | Y | 8 |
| Sanusi et al., (2021) | Y | N | N | Y | Y | Y | Y | N | N | N | 5 |
| Sediri et al., (2020) | Y | Y | N | Y | Y | Y | Y | Y | N | Y | 8 |
| Tadesse et al., (2020) | Y | Y | Y | Y | Y | Y | N | Y | N | Y | 8 |
| Tesfaye et al., (2021) | Y | Y | Y | Y | Y | Y | Y | N | Y | Y | 9 |
| Teshome et al., (2020) | Y | Y | N | Y | Y | Y | Y | N | Y | Y | 8 |
| Tobin et al., (2021) | Y | Y | N | Y | Y | Y | Y | Y | Y | N | 8 |
| Youssef et al., (2020) | Y | N | N | Y | Y | Y | N | Y | Y | N | 6 |

*Abbreviations:* Y, yes; N, no.

*Total:* Computed by summing the number of ‘yes’ scores obtained

*Questions:*

1. Is the source of information reported regarding anxiety and/or depression?

2. Were inclusion criteria reported?

3. Were exclusion criteria reported?

4. Was the time frame of recruitment reported?

5. Was the recruitment setting reported?

6. Were subjects consecutively recruited or population based?

7. Have the outcomes for anxiety and/or depression been tested for measurement properties?

8. Were all participants included in the analysis?

9. Has confounding been assessed and controlled for (subgroups analysis of multi variate analysis)?

10. Was response rate reported?


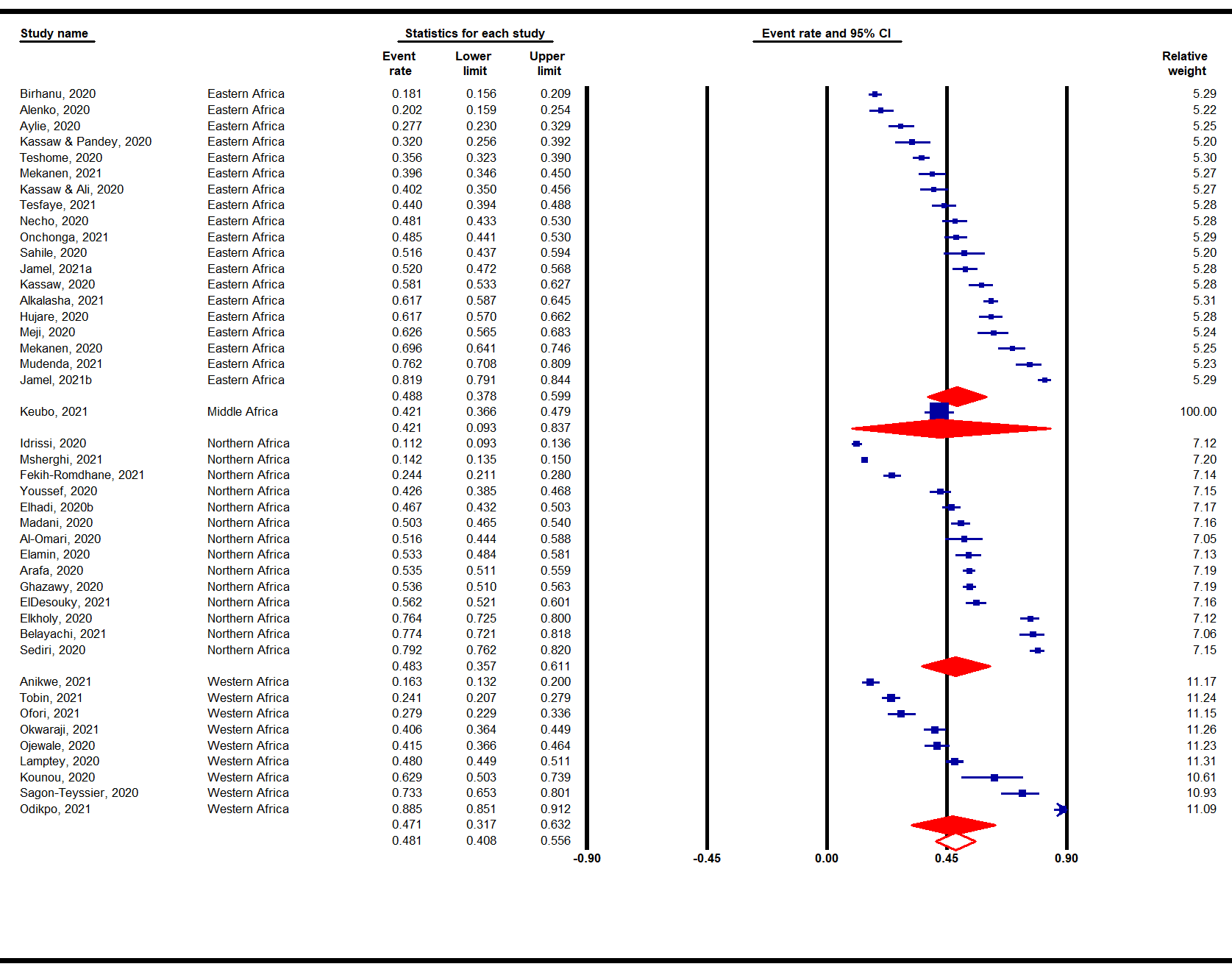
Appendix 3: Prevalence of anxiety in Africa by regions during the COVID-19 pandemic


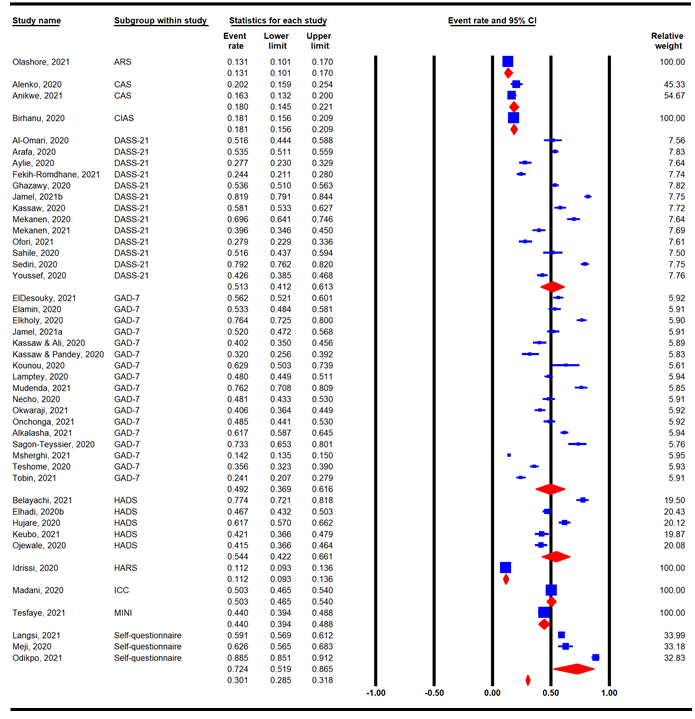
Appendix 4: Prevalence of anxiety in Africa by assessment tools utilized during the COVID-19 pandemic


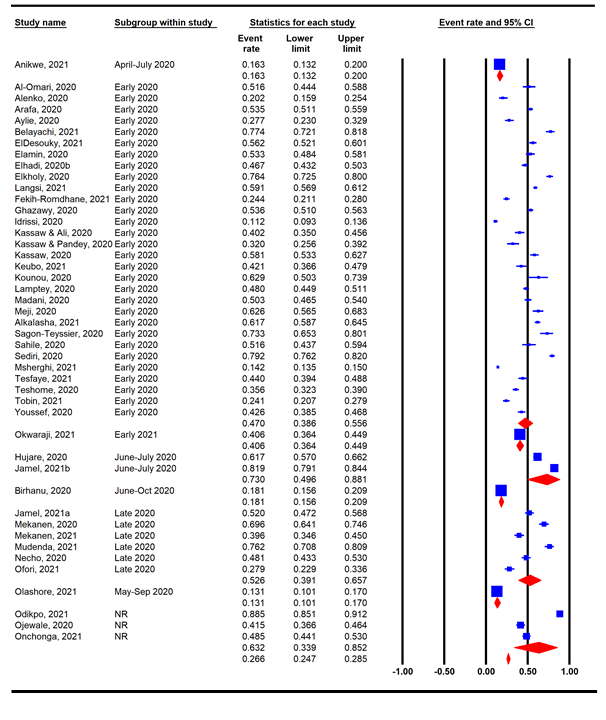


Appendix 5: Prevalence of anxiety in Africa by period of the COVID-19 pandemic


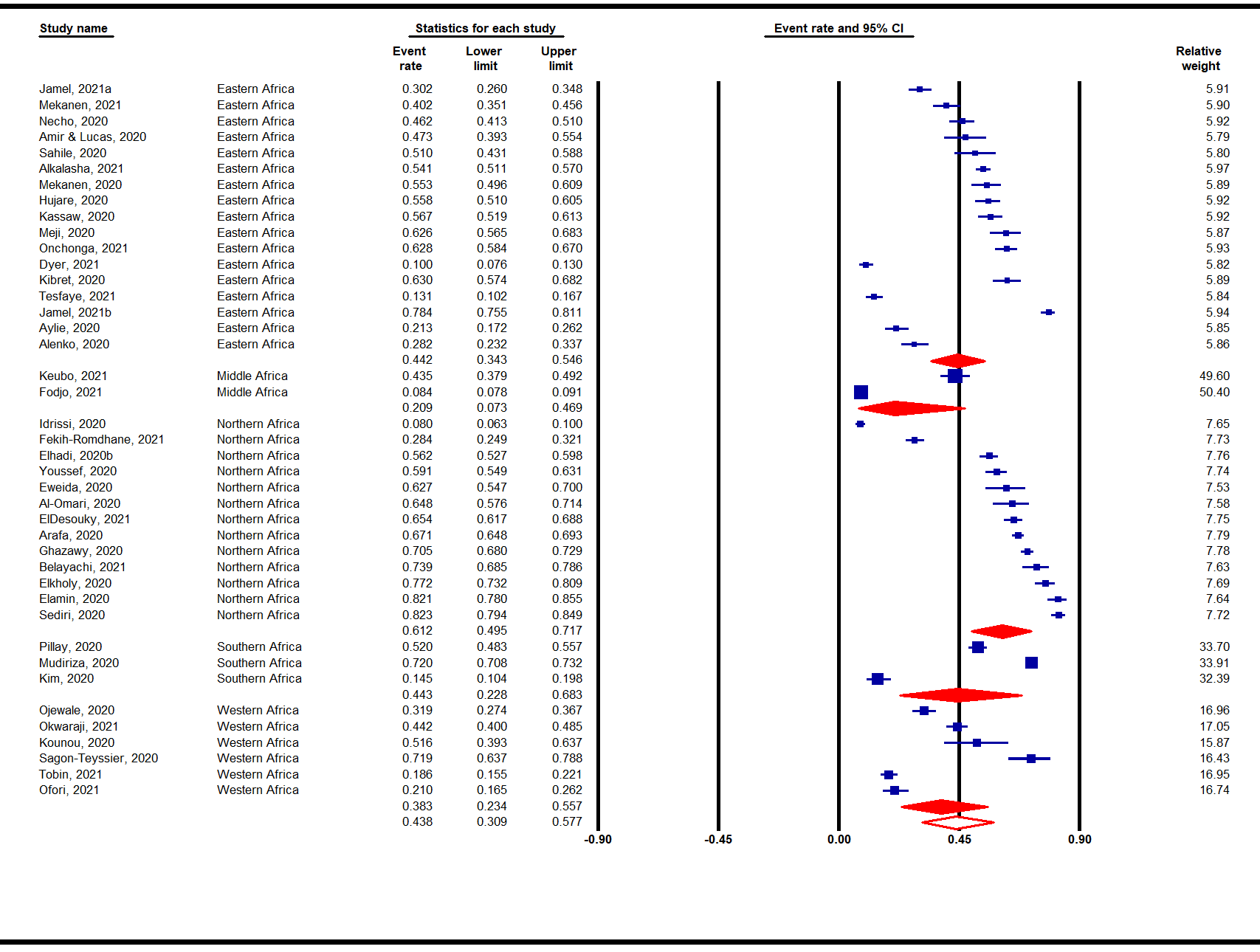


Appendix 6: Prevalence of depression in Africa by regions during the COVID-19 pandemic


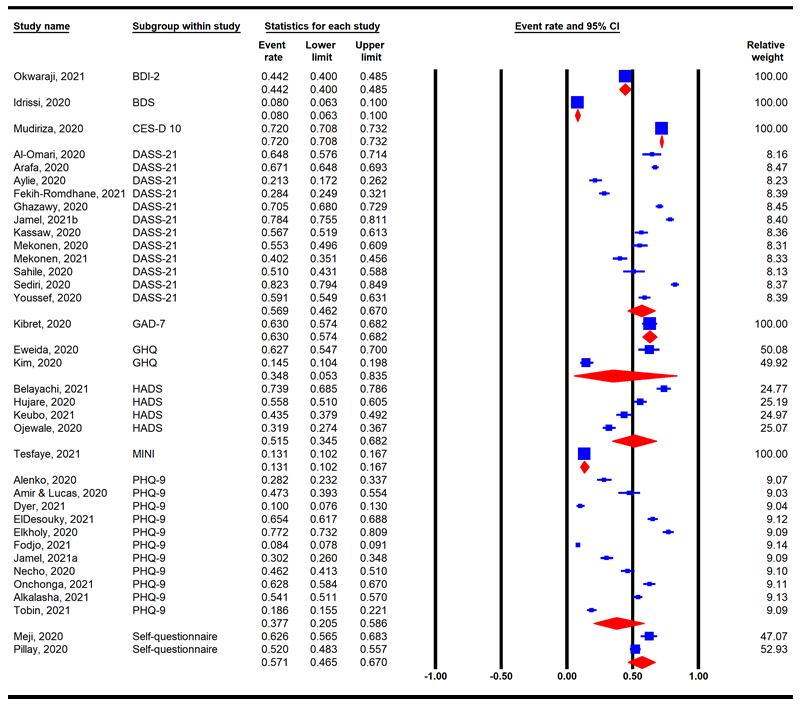


Appendix 7: Prevalence of depression in Africa by assessment tools utilized during the COVID-19 pandemic


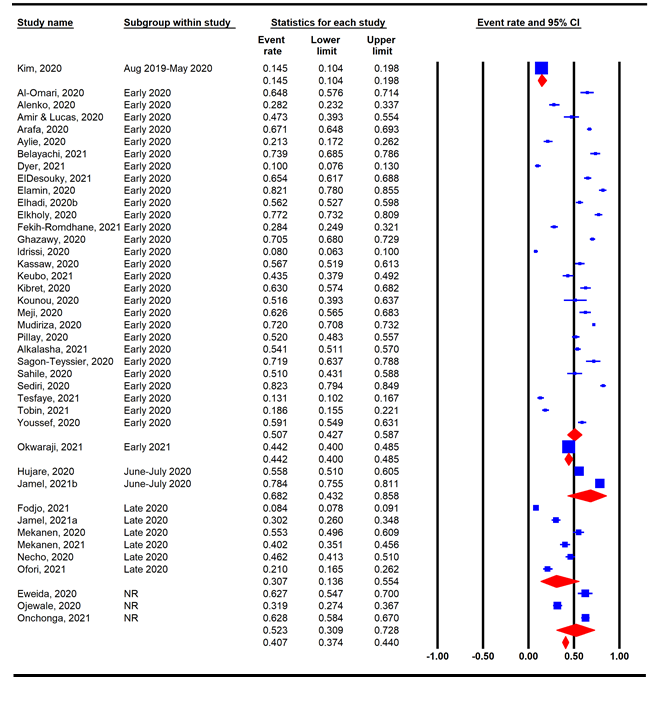


Appendix 8: Prevalence of depression in Africa by period of the COVID-19 pandemic
