# Supplementary material for: Prevalence of Anxiety and Depression Among the General Population in Africa During the COVID-19 Pandemic: A Systematic Review and Meta-Analysis
Source: Front Public Health. 2022 May 17;10:814981. doi: 10.3389/fpubh.2022.814981 (PMC9152218; doi:10.3389/fpubh.2022.814981)
Supplement: Supplementary file 2 [file Table_1.pdf]

Supplementary Table 1: Characteristics of included studies

Author	Study location	Study Period	Sample size	Gender	Age #range or mean age	Population	Sampling technique	Outcome measures used/Translated language	Outcome assessed
Abdelghani et al., (76)	Egypt	10th May-9th June 2020	N=320	**Frontline Physicians**: Male=21; female=27. **Second-line physicians**: Male=96; female=176	**Frontline Physicians**-Mean age: M= 32.3, SD= 4.5. **Second-line physicians**: Mean age: M=35.0, SD=6.2.	**Frontline Physicians** (n=48) **Second-line physicians** (n=272)	Systematic random method	HADS-Translated to Arabic/Psychometrically tested	Depression & anxiety
Afolabi (14)	Nigeria	NR	N=182	NR	Age range: 21-70; Mean age: M=5.16; SD=4.97	**Adults Residents**	Purposive sampling	GHQ-12 and GHQ-28/Not translated	Depression & anxiety
Agberotimi et al., (56)	Nigeria	20th March-19th April 2020	N=884	Male=482; female=402	Mean age: M=28.75; SD=8.17; Age range: 18-78 years	**Health workers** (382, 43.21)**;** **General residents** (n=502, 56.79%).	Snowball sampling	GAD-7 and PHQ-9/not translated	Depression and anxiety
Akorede et al., (90)	Nigeria	NR	N=137	NR	NR	**Nigerian students**	NR	No	Depression and anxiety
AlKalasha & Kasemy (68)	Ethiopia	March to May 2020	N=1,080	Male=384; Female=696	Mean age: M=29.98; SD=7.95.	**General population**	Convenience sampling	GAD-7/Arabic/ PHQ-9/Arabic/both psychometrically tested.	Depression and anxiety
Al Omari et al., (42)	Egypt	1st-15th April 2020	N=182	NR	Mean age: M=21.01; SD=1.69	**Youths aged 15-24years**	Online sampling	DASS-21 Scale/Arabic version/psychometricall y tested.	Depression and anxiety
Alenko et al., (39)	Ethiopia	15th May-14th June 2020	N=277	NR	Mean age: M=31.2; SD=4.80. Age range 23-43 years	**Healthcare professionals**	Online sampling	CAS/Not translated; PHQ-9/not translated	Depression and anxiety
Aluh et al., (91)	Nigeria	4th April-10th June 2020	N=1,011	Male=378; Female=633	Mean age: M=18.28; SD=5.9.	**General population**	Snowball sampling	COVID-19 Scale/Not translated	Anxiety
Alzueta et al., (36)	Sub-Saharan Africa	19th April- 3rd May 2020	N=83	NR	Mean age: M=42.30; SD=13.95	**Global population**	Snowball sampling	GAD-7 Scale and DASS-21/Translated and psychometrically tested to languages including French.	Depression and anxiety
Amir & Lucas (97)	Uganda	June 2020	N=146	Male=69; Female=77	NR	**Adult Refugees**	Convenience sampling	PHQ-9/not translated	Depression
Anikwe et al., (40)	Nigeria	April-July 2020	N=460	All females	Mean age: M=29.2; SD=NR	**Pregnant women**	Simple random sampling	CAS/not translated	Anxiety
Arafa et al., (113)	Egypt	16th-30th April 2020	N=1,629	Male=690; Female=939	784 subjects ≥ 30years	**General population**	Online snowball sampling	DASS-21/Arabic psychometrically evaluated	Depression, anxiety and stress
Arafa et al., (112)	Egypt	14th-24th April 2020	N=426	Male=214 Female=212	NR	**Frontline healthcare workers**	Online snowball sampling	DASS-21/Arabic/ psychometrically evaluated	Depression anxiety, and stress
Aylie et al., (43)	Ethiopia	15th May-15th June 2020	N=314	Male=199 Female=115	Mean age: M=22.58; SD: 2.8.	**University Students**	Systematic random sampling	DASS-21/Amharic/psychometri cally evaluated	Depression, anxiety, and stress
Belayachi et al., (27)	Morocco	23rd-30th March 2020	N=287	NR	NR	**Healthcare workers**	NR	HADS/not translated	Depression and anxiety
Birhanu et al., (41)	Ethiopia	June-October 2020	N=801	Male= 415 Female=386.	Mean age: M=34.8; SD=14.0	**Urban residents**	Multi-stage sampling	COVID-19 Induced Anxiety Scale/not translated	Anxiety
Boateng et al., (93)	Ghana	13th June-30th Sept 2020	N=811	Male=406; Female=405	Age ranged from 18-65+ years	**General population**	Convenience sampling	GAD-7 and WHO-5 Wellbeing Index/both not translated	Anxiety

Cénat et al., (15)	Rwanda, DRC, Haiti, and Togo	March-May 2020	N=1,267	Male=750<br>Female=517	Mean age: M=32; SD=10.1.	**General population**	Convenience sampling	HSCL/not translated	Anxiety
Cénat et al., (33)	Rwanda, DRC, Haiti, and Togo	March-May 2020	N= 1,267	Male=750<br>Female=517	Mean age: M=32; SD=10.1.	**General population**	Online sampling	HSC/translated to Creole, French, Kinyarwanda/psychometrically tested	Depression
Dyer et al., (32)	Kenya	March 2020	N=486	Male=171<br>Female=315	Age range: 10-24 years	**Adults living with HIV/AIDS**	NR	PHQ-9/not translated	Depression
Eisenbeck et al., (34)	Algeria, Egypt, and Nigeria	March-June 2020	N=992	Male=468<br>Female=524	Age range: 18-84 years	**Global population**	Online sampling	DASS-21/ not translated	Depression, anxiety, and stress
Ejeh et al., (84)	Nigeria	April 2020	N=346	Male=249<br>Female=97	Age range: 20-49 years	**Healthcare workers**	Online sampling	No	Anxiety
El Desouky et al., (58)	Egypt	1st-7th April 2020	N=1,040	Male=458<br>Female=582	Mean age: M=28.8; SD=7.7	**Egyptian adults**	Convenience & snowball sampling	GAD-7 and PHQ-9/Arabic/psychometrically tested	Depression and anxiety
Elamin et al., (57)	Sudan	Jan-March 2020	N=396	Male=124<br>Female=262	Majority 25 to 35years	**Healthcare workers**:<br>1. House officers (n=122)<br>2. Medical officers (n=93)<br>3. Registrars (n=53)<br>4. Specialist (n=21)<br>5. Consultant (n=19)<br>6. Nurses (n=21)<br>7. Lab Technicians (n=35)<br>8. Pharmacists (n=17)<br>9. Dentists (12)<br>10. Physiotherapists (n=1)<br>11. Radiology technicians (n=1), and<br>12. Health Officers (n=1).	Stratified random sampling	GAD-7 and PHQ-9 /Arabic/psychometrically tested	Depression and anxiety
Elhadi et al., (70)	Libya	20th April-1st May 2020	N=2,430	Male=1,919<br>Female=511	Mean age: M=23.30; SD=2.61	**Medical students**	Online sampling	GAD-7 and PHQ-9/not translated	Depression and anxiety
Elhadi et al., (79)	Libya	18th-28th April 2020	N=745	Male=358<br>Female= 387	Mean age: M=33.3; SD=7.4	**Healthcare workers**	Online sampling	HADS/not translated	Depression and anxiety
Elhadi et al., (77)	Libya	April 2020	N=154	Male=72<br>Female=82	Meana age: M=31.66; SD=5.97	**Frontline emergency physicians**	Online sampling	HADS/not translated	Depression and anxiety
Elkholy et al., (71)	Egypt	April and May 2020	N=502	Male=251<br>Female=251	Age range: 18 to ≥40 years	**Frontline healthcare workers**	Multi-stage probability sampling	GAD-7 and PHQ-9/Arabic/psychometrically tested	Depression, anxiety, and stress
El-Zoghby et al., (82)	Egypt	2nd-9th May 2020	N=502	Male=174<br>Female=336	Half of sample: 18 to 30years	**General Adult Population**	Convenience & snowball sampling	IES-R/Arabic/psychometrically tested	Stress and anxiety
Eweida et al., (94)	Egypt	NR	N=150	Male=47<br>Female=103	Age range: 23 to 24years	**Intern Nursing Students**	Simple random sampling	Middle East Respiratory Syndrome COVID-19 (MERS-COV) staff questionnaire/not translated	Stress and depression
Fekih-Romdhane & Cheour (44)	Tunisia	9th-15th April 2020	N=603	Male=157<br>Female=446	Age range: 18-65 years	**General Population**	Snowball sampling	DASS-21/Arabic/psychometrically tested	Depression, anxiety, and stress

Fodjo et al., (28)	Cameroon	5th June-5th Dec 2020	N=7,381	Male=5,409<br>Female=1,972	Mean age: M=30; IQR=25.0-38.0)	**General Population**	Online sampling	Fear of COVID-19 Scale and PHQ-9/French, not tested psychometrically	Depression
Ghazawy et al., (45)	Egypt	May 2020	N=1,335	Male=510<br>Female=825	Majority were 21 to 22 years	**University Students:**<br>1. Medical students (n=694)<br>2. Non-medical students (n=640).	Snowball sampling	DASS-21/Arabic/psychometric ally tested tool.	Depression, anxiety, and stress
Hajure et al., (78)	Ethiopia	1st June-30th July 2020	N=411	Male=217<br>Female=194	Mean age: M=43.3; SD=13.3	**Chronic Patients**	Consecutive sampling	HADS/not translated	Depression and anxiety
Idowu et al., (29)	Nigeria	22nd June-16th July 2020	N=1,010	Male=486<br>Female=524	Age range: 15-39 years	**Medical Students**	Purposive sampling	GHQ-12/not translated	Depression and anxiety
Idrissi et al., (80)	Morocco	1st April-1st May 2020	N=827	Male=395<br>Female=432	Mean age: M=35.9; SD=2.5	**General Population**	Online sampling	HARS and BDI/not translated	Depression and anxiety
Jemal et al., (59)	Ethiopia	July 2020	N=417	Male=279<br>Female=138	Majority 20 to 30 years	**Healthcare Workers**<br>1. Nurses (n=221)<br>2. Physicians (n=98)<br>3. Medical laboratory (n=98)	Simple random sampling	GAD-7 and PHQ-9/translated to Afam Omoro/Amharic/but not psychometrically tested in this context.	Depression, anxiety, and stress
Jemal et al., (46)	Ethiopia	25th June-25th July 2020	N=816	Male=540<br>Female=276	Majority 26 to 30 years	**Healthcare Workers**<br>1. Nurse (n=236)<br>2. Physicians (n=136)<br>3. Midwife (n=192)<br>4. Medical laboratory (68)<br>5. Health officers (n=96), and<br>6. Pharmacists (n=88)	Stratified random sampling	DASS-21/Translated to Afan Oromo & Amharic	Depression, anxiety, and stress
Kassawa & Ali (60)	Ethiopia	5th-10th April 2020	N=326	Male=133<br>Female=193	Majority were less than 35 years	**Healthcare workers**<br>1. Doctors (n=40)<br>2. Anaesthetist (n=16)<br>3. Laboratory scientist (n=26)<br>4. Pharmacist (n=28)<br>5. Psychiatrist (n=15)<br>6. Midwives (n=25)<br>7. Nurses (n=176).	Consecutive sampling	GAD-7/not translated	Anxiety
Kassawa & Pandey (61)	Ethiopia	6th April-6th May 2020	N=178	NR	Mean age: M=28; SD=5.6.	**Perinatal attending mothers**	Consecutive sampling	GAD-7/not translated	Depression, anxiety, and stress
Kassaw & Pandey (47)	Ethiopia	10th March-10th April 2020	N=420	Male=181<br>Female=230	≤28 and ≥2 8years	**General Population**	Multi-stage sampling	DASS-21/not translated	Anxiety
Kassaw (48)	Ethiopia	10th-30th March 2020	N=420	Male=189<br>Female=231	Mean age: M=27.0; SD=NR	**General Population**	Consecutive sampling	DASS-21/not translated	Depression, anxiety, and stress
Keubo et al., (75)	Cameroon	5th-19th April 2020	N=292	Male=159<br>Female=133	Age range: 20-60 years	**Healthcare Workers**<br>1. Laboratory assistants (n=2)<br>2. Nurse Assistants (n=19)<br>3. Nurses (168)<br>4. Doctors (74)<br>5. Clinical psychologists (n=5)<br>6. Medical laboratory technicians (n=18)	Convenience sampling	HADS/not translated	Depression and anxiety

						7. Other health technicians (6).			
Khalaf et al., (49)	Egypt	March-May 2020	N=170	Male=66<br>Female=104	Mean age: M=36.5; SD=5.08	**Medical Doctors**<br>1. Surgical specialists (n=44)<br>2. Medical specialists (n=87)<br>3. Supportive specialists (n=29).	Convenience sampling	DASS-21/Not translated	Depression, anxiety, and stress
Kibret et al., (95)	Ethiopia	15th May-15th June 2020	N=305	Male=104<br>Female=201	Almost half were 20 to 29 29years	**Healthcare Workers**	NR	GAD-7/not translated	Anxiety
Kim et al., (96)	South Africa	August 2019-May 2020	N=221	Male=59<br>Female=162	Mean age: M=46.3; SD=12.9	**Adults Population**	Simple random sampling	GHQ-28; CES-D/not translated	Depression and stress
Kounou et al., (62)	Togo	May 2020	N=62	Male=27<br>Female=35	Mean age: M=35.5; SD=8.75	**Medical Professionals**<br>1. Nurses (n=20)<br>2. Doctors (n=19)<br>3. Laboratory technicians (n=6)<br>4. Others (n=17)	NR	GAD-7/PSM-9 and PHQ-9/not translated	Depression, anxiety, and stress
Lamptey (38)	West African countries	1st April-31st May 2020	N=1000<br>1. Ghana=365<br>2. Nigeria=268<br>3. Benin=78<br>4. Togo=60<br>5. Senegal=46<br>6. Ivory Coast=40<br>7. Guinea=37<br>8. Liberia=31<br>9. Gambia=28<br>10. Sierra Leone=19<br>11. Burkina Fasso=16<br>12. Niger=14	Male=240<br>Female=760	Age range: Majority within 21-30 years	**Nurses**	Online sampling	GAD-7 and PSS-10/not translated	Anxiety and stress
Langsi et al., (37)	Sub-Saharan African Countries	17th April-17th May 2020	N=2005<br>Africans=1,855<br>Diaspora=150<br>1. West Africa=1,108<br>2. East Africa=209<br>3. Central Africa=251<br>4. Southern Africa=401	Male=1,099<br>Female=892	NR	**General Population**	Snowball sampling	No	Anxiety
Madani et al., (81)	Algeria	23rd March-12th April 2020	N=678	Male=405<br>Female=273	Age range: 14-74 years	**Confined population**	Snowball sampling	No	Stress and anxiety
Meji & Dennison (85)	Uganda	April-May 2020	N=253	Male=146<br>Female=106	NR	**Students**	Simple random sampling	No	Anxiety
Mekonen et al., (51)	Ethiopia	10th-30th Nov 2020	N=338	Male=190<br>Female=148	Mean age: M=24.70; SD=2.78	**University Students**<br>1. Health related discipline (n=64)<br>2. Non-health related (n=274)	Simple random sampling	DASS-21/not translated	Depression, anxiety, and stress
Mekonen et al., (50)	Ethiopia	25th Sept-20th Oct 2020	N=293	Male=160<br>Female=133	Mean age: M=29.6; SD=5.1	**Nurses**<br>1. Diploma (n=35)<br>2. BSc Nurses (n=175)<br>3. MSc Nurses (83)	Simple random sampling	DASS-21/not translated	Depression, anxiety, and stress

Moyer et al., (92)	Ghana	8th July-7th Aug 2020	N=71	NR	Age range: <20 to >40years	**Pregnant Women**<br>1. Urban=47<br>2. Peri-urban=13<br>3. Rural=9	Online sampling	No	Anxiety
Msherghi et al., (72)	Libya	May-June 2020	N=8,084	Male=2,994<br>Female=5,090	Mean age: M=27.2; SD=8.9	**General Population**	Online sampling	GAD-7/not translated	Anxiety
Mudenda et al., (65)	Zambia	August-Sept 2020	N=273	Male=132<br>Female=141	Median age=24; IQR (22-27)	**Pharmacy students**	Online sampling	GAD-7/not translated	Anxiety
Mudiriza & De Lannoy (30)	South Africa	29th April-21st May 2020	N=5,693	Male=3,472<br>Female=2,221	Age range: 18-35 years	**General population**	Online sampling	CES-D/not translated	Depression
Necho et al., (66)	Ethiopia	15th-30th July 2020	N=403	Male=239<br>Female=164	Mean age: M=36.66; SD=6.09	**People with disabilities**	Purposive sampling	GAD-7 and PHQ-9/not translated	Depression and anxiety
Odikpo et al., (86)	Nigeria	NR	N=418	Male=78<br>Female=340	Mean age: M=37.81; SD=8.21	**Nurses**	Multi-stage sampling	No	Anxiety
Ofori et al., (52)	Ghana	11th July-12th Aug 2020	N=236	Male=115<br>Female=121	Mean age: M=30.2; SD=5.2	**Healthcare Workers**	Convenience sampling	DASS-21/not translated	Anxiety
Ojewale (31)	Nigeria	NR	N=386	Male=154<br>Female=232	Mean age: M=21; SD=2.9	**Students**	Convenience sampling	HADS/not translated	Depression, anxiety, and stress
Okwaraji & Onyebueke (63)	Nigeria	Jan-Feb 2021	N=520	Male=260<br>Female=280	Mean age: M=37.05; SD=7.35	**Urban Dwellers**	Simple random sampling	GAD-7 and BDI/not translated	Depression and anxiety
Olaseni et al., (64)	Nigeria	20th March-12th April 2020	N=502	Male=269<br>Female=225<br>Undisclosed sex=8	Mean age: M=28.75; SD=8.17	**General Population**	Snowball sampling	GAD-7 and PHQ-9/not translated	Depression
Olashore et al., (35)	Nigeria and Botswana	1st May-30th Sept 2020	N=373	Male=130<br>Female=242	Mean age: M=38.42; SD=8.10	**Healthcare Workers**	NR	ARS/not translated	Anxiety
Onchonga et al., (67)	Kenya	NR	N=476	Male=231<br>Female=254	NR	**Healthcare Workers**	Stratified sampling	GAD-7 and PHQ-9/not translated	Anxiety and depression
Pillay et al., (98)	South Africa	28th-30th April 2020	N=692	Male=463<br>Female=225	NR	**Athletes**	Convenience sampling	No	Depression
Rakhmanov & Dane (87)	Nigeria	NR	N=183	Male=69<br>Female=114	Mean age: Men: M=19.71; SD=2.69; Women: M=19.0; SD=2.49	**University Students**	Online sampling	No	Anxiety
Rakhmanov et al., (88)	Nigeria	NR	N=69	Male=49<br>Female=20	Age range: 17-21 years	**University Staff**	Online sampling	SRQ-20/not translated, and not tested psychometrically	Depression and anxiety
Rakhmanov et al., (89)	Nigeria	NR	N=287	Male=151<br>Female=136	NR	**Secondary school students**	NR	GAD-7/not translated	Anxiety
Sagaon-Teyssier et al., (69)	Mali	6th-11th April 2020	N=135	Male=82<br>Female=53	Median age: 40 years; IQR (33-46)	**Healthcare Workers**<br>1. Doctors/pharmacist/ midwifes (n=35)<br>2. Nurses (n=20)<br>3. CHW or Psychosocial counsellor (n=52)<br>4. Others (n=28)	NR	GAD-7 and PHQ-9/not translated	Depression and anxiety
Sahile et al., (54)	Ethiopia	27th April-7th May 2020	N=153	Male=47<br>Female=106	Age range: 22-24 years	**Students**	Online sampling	DASS-21/not translated	Depression, anxiety and stress
Sanusi et al., (99)	Nigeria	6th-18th April 2020	N=900	Male=444<br>Female=456	NR	**General Population**	Purposive sampling	No	Depression
Sediri et al., (55)	Tunisia	25th April-6th May 2020	N=751	Female=751	Mean age: M=37; SD=8.2	**Women Population**	Snowball sampling	DASS-21/Arabic/psychometrically tested	Depression, anxiety and stress

Tadesse et al., (100)	Ethiopia	March-April 2020	N=415	Male=174<br>Female=241	Mean age: M=30; SD=3	**Nurses**	Simple random sampling	Psychological response towards the COVID-19 Outbreak/not translated	depression
Tesfaye et al., (83)	Ethiopia	1st-22nd March 2020	N=420	Male=190<br>Female=230	Mean age: M=37.2; SD=11.9	**General Population**	Systematic random sampling	M.I.N.I-5.0.0/not translated	Depression and anxiety
Teshome et al., (73)	Ethiopia	20th May-20th June 2020	N=798	Male=482<br>Female=316	Mean age:<br>M=29.29; SD=5.69	**Healthcare Workers**<br>1. Clinical Nurse (n=356)<br>2. Medical Doctors (n=65)<br>3. Medical Laboratory (n=84)<br>4. Midwifery (n=120)<br>5. Pharmacist (n=77)<br>6. Public Health Officers (n=96)	Simple random sampling	GAD-7/not translated	Anxiety
Tobin et al., (74)	Nigeria	May-June 2020	N=543	Male=256<br>Female=287	Mean age: M=27.7; SD=9.38	**General Population**	Convenience sampling	GAD-7 and PHQ-9/not translated	Depression and anxiety
Youssef et al., (53)	Egypt	April 2020	N=540	Male=294<br>Female=246	Mean age:<br>M=37.3; SD=9.2	**Healthcare Workers**<br>1. Physicians (n=416)<br>2. Nursing (n=49)<br>3. Pharmacist (38)<br>4. House Officers (n=12)<br>5. Technicians (n=9)<br>6. Others (n=16)	Snowball sampling	DASS-21/Arabic/psychometrically tested	Depression, anxiety and stress
**Total**			62,380	Male=31,074<br>Female=29,479					

Abbreviations: ARS: Anxiety Rating Scale; BDI: Beck Depression Inventory; CAS: Coronavirus Anxiety Scale; DASS-21: Depression Anxiety Stress Scale-21; Generalized Anxiety Disorder-7; GHQ-28: General Health Questionnaire-28; HADS: HARS: Hamilton Anxiety Rating Scale; Hospital Anxiety and Depression Scale; HSC: Hopkins Symptoms Checklist; IES-R: Impact of Event Scale-Revised; IQR: Inter Quantile Range; M.I.N.I-5.0.0 Mini-International Neuropsychiatric Interview; M: Mean; NR: Not Reported; No=Authors developed a questionnaire with no psychometric testing; PHQ-9: Patient Health Questionnaire; PSM-9: Psychological Stress Measure; PSS-10: Perceived Stress Scale-10; STAI: State-Trait Anxiety Inventory; SD: Standard Deviation; Short Mood & Feelings Questionnaire; SRQ-20: Self-Reported Questionnaire; SDS: Self-rating Depression Scale and WHO: World Health Organization.
